# Differential expression of *SlKLUH* controlling fruit and seed weight is associated with changes in lipid metabolism and photosynthesis-related genes

**DOI:** 10.1093/jxb/eraa518

**Published:** 2020-11-07

**Authors:** Qiang Li, Manohar Chakrabarti, Nathan K Taitano, Yozo Okazaki, Kazuki Saito, Ayed M Al-Abdallat, Esther van der Knaap

**Affiliations:** 1 State Key Laboratory of North China Crop Improvement and Regulation, Key Laboratory of Vegetable Germplasm Innovation and Utilization of Hebei, Collaborative Innovation Center of Vegetable Industry in Hebei, College of Horticulture, Hebei Agricultural University, Baoding, China; 2 Center for Applied Genetic Technologies, University of Georgia, Athens, GA, USA; 3 Department of Plant and Soil Sciences, University of Kentucky, Lexington, KY, USA; 4 Institute for Plant Breeding, Genetics and Genomics, University of Georgia, Athens, GA, USA; 5 RIKEN Center for Sustainable Resource Science, Yokohama, Japan; 6 Graduate School of Bioresources, Mie University, Tsu, Japan; 7 Graduate School of Pharmaceutical Sciences, Chiba University, Chiba, Japan; 8 School of Agriculture, The University of Jordan, Amman, Jordan; 9 Department of Horticulture, University of Georgia, Athens, GA, USA

**Keywords:** Fruit weight, KLUH, lipid metabolism, seed weight, tomato, transcription factor

## Abstract

The sizes of plant organs such as fruit and seed are crucial yield components. Tomato KLUH underlies the locus *fw3.2*, an important regulator of fruit and seed weight. However, the mechanism by which the expression levels of *KLUH* affect organ size is poorly understood. We found that higher expression of *SlKLUH* increased cell proliferation in the pericarp within 5 d post-anthesis in tomato near-isogenic lines. Differential gene expression analyses showed that lower expression of *SlKLUH* was associated with increased expression of genes involved in lipid metabolism. Lipidomic analysis revealed that repression of *SlKLUH* mainly increased the contents of certain non-phosphorus glycerolipids and phospholipids and decreased the contents of four unknown lipids. Co-expression network analyses revealed that lipid metabolism was possibly associated with but not directly controlled by *SlKLUH*, and that this gene instead controls photosynthesis-related processes. In addition, many transcription factors putatively involved in the KLUH pathway were identified. Collectively, we show that SlKLUH regulates fruit and seed weight which is associated with altered lipid metabolism. The results expand our understanding of fruit and seed weight regulation and offer a valuable resource for functional studies of candidate genes putatively involved in regulation of organ size in tomato and other crops.

## Teaser

*KLUH* expression was found to be associated with lipid metabolism and photosynthesis in tomato, leading to novel insights into organ growth.

## Introduction

The weight/size of plant organs is critically important for the survival of the species (Gonzalez *et al.*, 2009). The final weight of a plant organ is influenced by the combined effect of genetic and environmental signals during growth and development of the plant (Tsukaya, 2003; Horiguchi *et al.*, 2005). The weight of organs such as seed, fruit, root, tuber, and leaf is of importance to plant yield as this is one of the most critical agronomic traits in crop breeding (Ge *et al.*, 2016).

The regulation of seed and leaf size has been studied extensively in rice and Arabidopsis, respectively. These studies have led to the discovery of at least 88 key organ size regulators (Gonzalez *et al.*, 2009; Li and Li, 2016; Vercruysse *et al*., 2020). The pathways that control seed size include KLUH, ubiquitin–proteasome, G-protein signaling, mitogen-activated protein kinase (MAPK), and plant hormones (Gonzalez *et al.*, 2009; Li and Li, 2016; Li *et al.*, 2019). For leaf size, in addition to KLUH, the pathways that control this trait are DA1–enhancer of DA1 (EOD1), growth regulating factor (GRF)–GRF-interacting factor (GIF), SWITCH/sucrose non-fermenting (SWI/SNF), and plant hormones (Vercruysse *et al*., 2020). Fruit weight is most extensively studied in tomato (van der Knaap *et al.*, 2014; Mu *et al.*, 2017). The pathways regulating fruit weight are also KLUH, as well as cell number regulator (CNR), cell size regulator (CSR), members of the WUS–CLV3 pathway, and plant hormones (van der Knaap and Østergaard, 2018; Rothan *et al.*, 2019). Remarkably, one of the shared components in seed, leaf, and fruit size regulation is KLUH. However, the role of KLUH and its relationship to other organ size regulatory pathways is not well understood.

KLUH is the founding member of the CYP78A subfamily that was first identified in Arabidopsis to stimulate organ size by promoting cell proliferation (Anastasiou *et al.*, 2007; Adamski *et al.*, 2009). KLUH is proposed to be involved in the production of an unknown signaling molecule that non-cell-autonomously regulates cell proliferation (Anastasiou *et al.*, 2007; Adamski *et al.*, 2009; Eriksson *et al.*, 2010). However, the exact molecular and biochemical nature of the mobile signal remains unknown. Notably, other members of the CYP78A subfamily are also associated with controlling organ size in Arabidopsis (Wang *et al.*, 2008; Fang *et al.*, 2012; Sotelo-Silveira *et al.*, 2013; Yang *et al.*, 2013) as well as in other plant species (Ma *et al*., 2013; Nagasawa *et al.*, 2013; Yang *et al.*, 2013; Ma *et al*., 2015*a*, *b*; Wang *et al.*, 2015*a*; Zhao *et al.*, 2016; X. Sun *et al.*, 2017; Qi *et al.*, 2017; Maeda *et al.*, 2019). In rice, *GIANT EMBRYO* (*GE*; *CYP78A13*) plays an important role in controlling the size balance of the embryo and endosperm. This gene is essential for embryo development and grain yield (Nagasawa *et al.*, 2013; Yang *et al.*, 2013). The rice CYP78A *OsBSR2* (*BROAD- SPECTRUM RESISTANCE2*) is associated with seed weight and disease resistance (Maeda *et al.*, 2019). The maize *CYP78A PLASTOCHRON1* (*ZmPLA1*) extends the duration of cell division, leading to increased seed yield and stover biomass (X. Sun *et al.*, 2017). In soybean, wheat, sweet cherry, and pepper, *GmCYP78A10*, *GmCYP78A72*, *TaCYP78A3*, *TaCYP78A5*, *PaCYP78A9*, and *CaKLUH*, respectively, play important roles in or are strongly associated with regulating seed and fruit weight (Chakrabarti *et al.*, 2013; Ma *et al*., 2013; Ma *et al*., 2015*a*, *b*; Wang *et al.*, 2015*a*; Zhao *et al.*, 2016). Combined, these studies demonstrate the importance of CYP78A as a critical component of organ size regulation in plants.

The domestication-related *CYP78A* gene was cloned from tomato a few years ago and considered the ortholog of Arabidopsis *KLUH* (Zhang, 2012; Chakrabarti *et al.*, 2013). Tomato *KLUH* underlies the fruit weight locus *fw3.2* and is a positive regulator of fruit weight by increasing the number of cell layers in the pericarp (Chakrabarti *et al.*, 2013). We recently demonstrated that the duplication of *SlKLUH* is the causative variant at the *fw3.2* locus, accounting for differential expression that is correlated to gene copy number (Alonge *et al.*, 2020). Given that *SlKLUH* does not affect cell size (Chakrabarti *et al.*, 2013), it is likely to function in the cell proliferation phase in pericarp at the early stages of fruit development. However, further cellular analyses at different fruit developmental stages are needed to determine when changes in the number of cell layers become evident.

In this study, we performed histological comparisons of *fw3.2* near-isogenic lines (NILs) to investigate the changes in the number of cell layers in the pericarp at six developmental time points. We analyzed the RNA sequencing (RNA-seq) data from developing pericarp and seed in *fw3.2* NILs that only differ for the allele at the locus as well as lines that are transgenically down-regulating the expression of *SlKLUH* by RNAi (*RNAi-2Q1*). The results showed many differentially expressed and co-regulated genes that have been implicated in organ size, lipid metabolism, and photosynthesis. We also analyzed the lipid profiles of 5 days post-anthesis (DPA) fruits from the NILs and *RNAi-2Q1* and identified several lipid composition categories that were differentially accumulating. Moreover, the overexpression of a transcription factor (TF) gene *SHINE1* (*SlSHN1*) that affects lipid metabolism, resulted in a significant decrease in fruit and seed weight. Combined, our findings imply a tight relationship between SlKLUH-mediated regulation of organ weight and lipid metabolism as well as photosynthesis-related processes.

## Materials and methods

### Plant materials and growth conditions

NILs with the cultivated and wild-type allele of *fw3.2*, named *fw3.2*(*ys*) and *fw3.2*(*wt*), respectively, RNAi lines down-regulating the expression of *SlKLUH* (*RNAi-2Q1* and *RNAi-2G2*), and *SlSHN1*-overexpressing transgenic lines were described previously (Zhang, 2012; Chakrabarti *et al.*, 2013; Al-Abdallat *et al.*, 2014). The seeds of the *cd2* mutant and Ailsa Craig (AC) control were obtained from Dr Cornelius Barry, Michigan State University (Nadakuduti *et al.*, 2012). The plants were grown in the greenhouse under a 16 h light/8 h dark photoperiod in Athens, GA, USA.

### Developing fruit analyses for *fw3.2*(*ys*) and *fw3.2*(*wt*)

Individual flowers were tagged at anthesis every morning. Developing fruits were collected at anthesis, 5, 7, 10, and 20 DPA, and breaker stage. Developing fruits were bisected equatorially. One half of each fruit was scanned for fruit length and width measurement using ImageJ, and the other half was used for histological analysis.

For the histological analysis of ovaries and developing fruits at 5 and 7 DPA, the samples were fixed overnight in 75% ethanol and 25% acetic anhydride. Samples were then incubated in 80% ethanol at 80 °C and rehydrated in 50% and 30% ethanol for 10 min. Samples were rinsed with ddH_2_O for 10 min, followed by clearing at room temperature in 0.2 M NaOH/1% SDS while shaking at 30–40 rpm. After 24 h, the samples were further cleared with ClearSee solution (10% xylitol, 15% sodium deoxycholate, 25% urea; VWR International) for 3 d at the same shaking speed and temperature. The samples were rinsed with ddH_2_O for 5 min and stained for 30 min in calcofluor (0.25% Fluorescent Brightener 28; Sigma) in the dark. Lastly, the samples were rinsed in water and mounted in mounting medium CitiFlour (Electron Microscopy Supplies). The sections were imaged using a Zeiss LSM 880 upright confocal microscope and samples were excited at 405 nm with an emission band of 410–550 nm.

For developing fruits at 10 DPA, 20 DPA, and breaker stage, hand sections were stained with a solution containing one part 0.5% toluidine blue and two parts distilled water for a few seconds. Sections were then rinsed with ddH_2_O. Images of the stained sections were taken using an Olympus DP70 camera that was mounted on an OLYMPUS MVX10 optical microscope using an Olympus MVX-TVO.63XC adaptor. The generated pictures were used for pericarp cell layer, maximum cell size, and thickness measurements with ImageJ software as previously described (Ramos, 2018). All phenotypic evaluations were performed with two biological replications, each with at least four plants per genotype. For each time point, at least two fruits per plant were analyzed.

### Phenotypic evaluations of *SlSHN1*-overexpressing transgenic lines

For fruit weight analysis, 10 fruits at breaker or turning stage from each plant were weighed individually. For seed weight analysis, 50 seeds from each plant were counted and weighed. Three fruits at breaker stage from each plant were used for pericarp cell layer and thickness analysis as previously described (Ramos, 2018). All phenotypic evaluations were performed independently with two biological replications, each with at least three plants per genotype.

### Tissue collection and data processing of RNA-seq data

Tissues for RNA extraction were collected with four replicates from pericarp and seed at 5, 7, and 10 DPA in *fw3.2* NILs and three replicates from pericarp and seed at 7 DPA of the *RNAi-2Q1* and *RNAi-2G2* lines down-regulating *SlKLUH*. RNA-seq library preparation and sequencing were previously described (Chakrabarti *et al.*, 2013). All clean reads for samples from *fw3.2* NILs and the RNAi lines of *SlKLUH* are available in the National Center for Biotechnology Information Sequence Read Archive (NCBI SRA) under the accession numbers SRA068200 and SRA068201 (Chakrabarti *et al.*, 2013).

The read mapping was performed using the latest version of the Tuxedo protocol with HISAT2 and StringTie (Pertea *et al.*, 2016). After filtering out adaptor sequences, low-quality reads, and ribosomal reads, the clean reads from each library were mapped to the Heinz 1706 tomato genome version SL3.0 using HISAT2. To quantify all the genes in ITAG (International Tomato Annotation Group) version 3.20, the mapping results were normalized via Stringtie to obtain RPKM (reads per kilobase per million mapped reads). Summary statistics for each of the RNA-seq libraries are shown in Supplementary Table S1. Correlations between samples were determined by using the Spearman correlation coefficient (SCC) to check the reproducibility among replicates. For principal component analysis (PCA) of sample replicates, the count data were rlog transformed using DESeq2 and the PCA plot was generated using the ggplot2 R package.

### Differential gene expression analysis

Differential gene expression analysis was performed using the DESeq2 R package (Love *et al.*, 2014) with the count data which were extracted with a Python script included in Stringtie (http://ccb.jhu.edu/software/stringtie/dl/prepDE.py). The genes that were significantly differentially expressed in pericarp and seed at each developmental time point between the NILs as well as in 7 DPA pericarp and seed between *fw3.2*(*ys*) and *RNAi-2Q1* were identified by Wald test. Genes with |log_2_ratio|>2 and a false discovery rate (FDR) significance score <0.05 were determined to be significantly differentially expressed genes (DEGs). A differential expression analysis of RNA-seq data from the NILs was also performed using linear factorial modeling to further assess the effects of genotype, the interaction between genotype and developmental stage (G×D), and the interaction between genotype and tissue (G×T) on the gene expression patterns. The likelihood ratio test was used to assess three separate null hypotheses. Null hypothesis 1 was tested to identify genes with significant genotype effects with full model=~genotype+tissue+developmental stages and reduced model=~tissue+developmental stages; Null hypothesis 2 was tested to identify genes significantly affected by G×D with full model=~genotype+tissue+developmental stages+genotype:developmental stages and reduced model=~genotype+tissue+developmental stages; Null hypothesis 3 tested whether each gene was affected by G×T with full model=~genotype+tissue+developmental stages+genotype:tissue and reduced model=~genotype+tissue+developmental stages. The *P*-values were corrected using the Benjamini–Hochberg method, and the threshold of corrected *P*-value <0.05 was used for selecting DEGs in the three null hypotheses. A further filtration was performed to eliminate the genes expressed at a low level. Genes with average RPKM>1 among pericarp and seed samples were considered as DEGs. Additionally, the linear factorial modeling can only be applied to sufficiently large data sets with multiple treatments or time points. Since there is only one developmental time point in *RNAi-2Q1*, we cannot perform the linear factorial modeling with that specific dataset.

### Lipid profiling

Lipid profiling was done at RIKEN, Japan, using LC–quadrupole time-of-flight–MS (LC-Q-TOF-MS) as described before (Okazaki *et al.*, 2013; Okazaki and Saito, 2018). Briefly, 5 DPA whole fruit samples from *fw3.2*(*ys*), *fw3.2*(*wt*), and *RNAi-2Q1* were pooled from four plants each, and each sample was replicated five times. These samples were lyophilized and milled to a fine power. The sample powder was extracted with a mixture of chloroform, methanol, and water by the method of Bligh and Dyer (Bligh and Dyer, 1959; Okazaki and Saito, 2018). The crude lipid extract was finally reconstituted in ethanol and subjected to LC-MS analysis (Okazaki and Saito, 2018). Electrospray ionization was employed for sample ionization. The lipidome dataset obtained in the negative ion mode was subjected to multivariate analysis, orthogonal projection to a latent structure-discriminant analysis (OPLS-DA) (Wiklund *et al.*, 2008), to find the discriminative metabolites among tested samples.

### Construction and visualization of the co-expression network

The co-expression network analysis was performed in R using the Weighted Correlation Network Analysis (WGCNA) package (Langfelder and Horvath, 2008). The co-expression network was constructed using RNA-seq data from each NIL independently. For each co-expression network, the genes (cumulative RPKM >6 and variance >1) used for the network were from 24 samples of three time points (5, 7, and 10 DPA), using each biological replicate as an individual dataset (total of 24 samples for each network). To show an approximate scale-free topology, the soft thresholding power of β=17 was chosen for both networks by the pickSoftThreshold function in the WGCNA package. The modules were obtained using the one-step network construction function (blockwiseModules) with default parameters. The top 50 genes with the highest *k*_ME_ values were regarded as intramodular hub genes in this study. The networks were visualized using Cytoscape _v.3.7.1.

### Gene Ontology (GO) enrichment analysis

GO enrichment analysis of the DEGs was performed using the topGO R package (Alexa *et al.*, 2006; Alexa and Rahnenfuhrer, 2020). The reference GO annotation list was downloaded from Plant Transcriptional Regulatory Map (http://plantregmap.gao-lab.org/go.php). The significantly enriched GO terms were determined by FDR-adjusted *P*-value <0.05. The heatmaps of DEGs and GO terms were generated using the pheatmap R package (Kolde, 2012).

### Statistical analyses

Normality, Student’s *t*-test, and Duncan’s test were calculated for each trait using R software. The data of all the investigated traits follow a normal distribution as determined by Lilliefors test (Abdi and Molin, 2007).

## Results

### Fruit growth and histological comparisons of *fw3.2* NILs

**Fig. 1.**
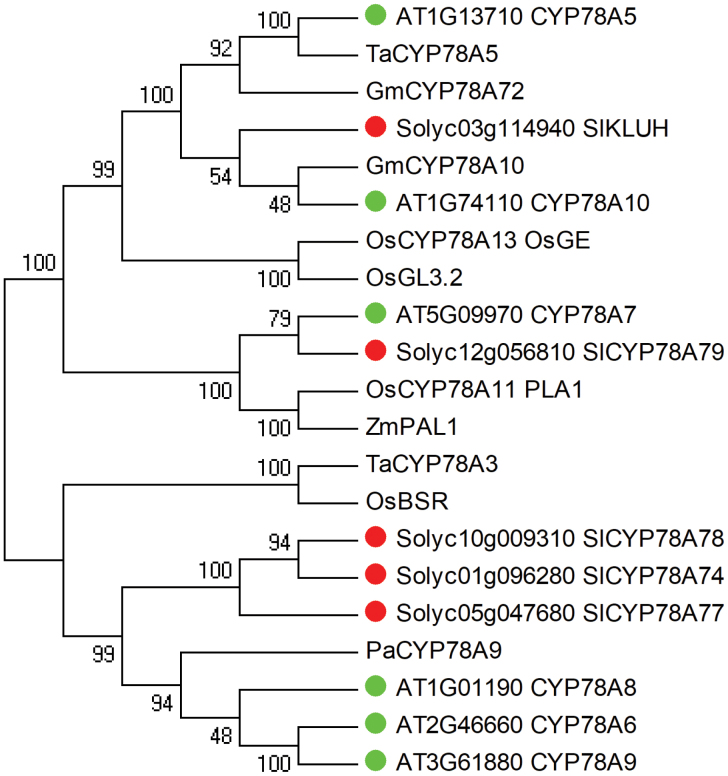
Phylogenetic analysis of CYP78As from tomato, Arabidopsis, rice, wheat, soybean, maize, and sweet cherry. The alignment of protein sequences was performed using ClustalX 1.81, and the phylogenetic tree was constructed by MEGA4 using the neighbor–joining (NJ) method with the following parameters: Poisson correction, pairwise deletion, and bootstrap (1000 replicates; random seed). Tomato and Arabidopsis CYP78As are labeled with red and green dots, respectively.

**Fig. 2.**
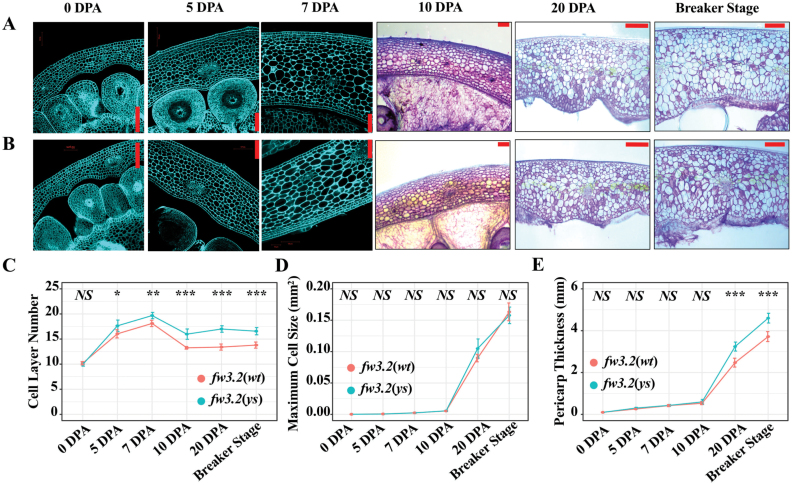
Histological analyses of the pericarp at six developmental time points in the *fw3.2* NILs. (A) Representative sections of *fw3.2*(*ys*) pericarp. (B) Representative sections of *fw3.2*(*wt*) pericarp. Scale bars=100 µm (0, 5, 7, and 10 DPA) and 1 mm (20 DPA and breaker stage). (C–E) Cell layer (C), maximum cell size (D), and pericarp thickness (E) comparisons of the NILs. For the cell layer numbers at 10 DPA, 20 DPA, and breaker stage, the endoderm layer and several cell layers below the exoderm were not counted because they were difficult to discern in these sections, hence a decrease in cell layers from 7 to 10 DPA. Asterisks denote significant differences (*P<0.05; **P<0.01; ***P<0.001) as determined by Student’s *t*-tests. DPA, days post-anthesis. NS, non-significant difference.

Phylogenetic analysis revealed that SlKLUH was clustered into the same clade with Arabidopsis CYP78A5/KLUH and CYP78A10, wheat CYP78A5, and soybean CYP78A10 and CYP78A12 (Fig. 1) that act as key regulators of organ size (Anastasiou *et al.*, 2007; Adamski *et al.*, 2009; Yang *et al.*, 2013; Ma *et al.*, 2015*b*; Wang *et al.*, 2015*a*; Zhao *et al.*, 2016). Of these, AtCYP78A5 and TaCYP78A5 have been demonstrated to stimulate cell proliferation at the early stages of seed development (Anastasiou *et al.*, 2007; Adamski *et al.*, 2009; Ma *et al.*, 2015*b*). To gain more insight into the function of SlKLUH in regulating fruit development, we explored fruit growth at six developmental time points from anthesis to breaker stage (Supplementary Fig. S1). Both fruit length and width started to show differences between 10 and 20 DPA (Supplementary Figs S1, S2A, B). This change in length and width was preceded by a significant change in the number of cell layers as early as 5 DPA (Fig. 2A–C; Supplementary Fig. S2C). The maximum cell size showed no significant difference between the NILs at breaker stage (Fig. 2D; Supplementary Fig. S2D) which is consistent with previous findings (Chakrabarti *et al.*, 2013). The pericarp thickness started to show a significant difference between 10 and 20 DPA which corresponded well to the increase in fruit size (Fig. 2E; Supplementary Fig. S2E). The significant difference in cell layers of the pericarp (Fig. 2C; Supplementary Fig. S2C) did not lead to a significant difference in pericarp thickness at 5, 7, and 10 DPA (Fig. 2E; Supplementary Fig. S2E), which was probably due to the small cell size (Fig. 2D; Supplementary Fig. S2D). These results indicate that SlKLUH stimulates pericarp cell proliferation during the early stages of fruit development (5–7 DPA).

### Differential gene expression between the NILs during pericarp and seed development

**Fig. 3.**
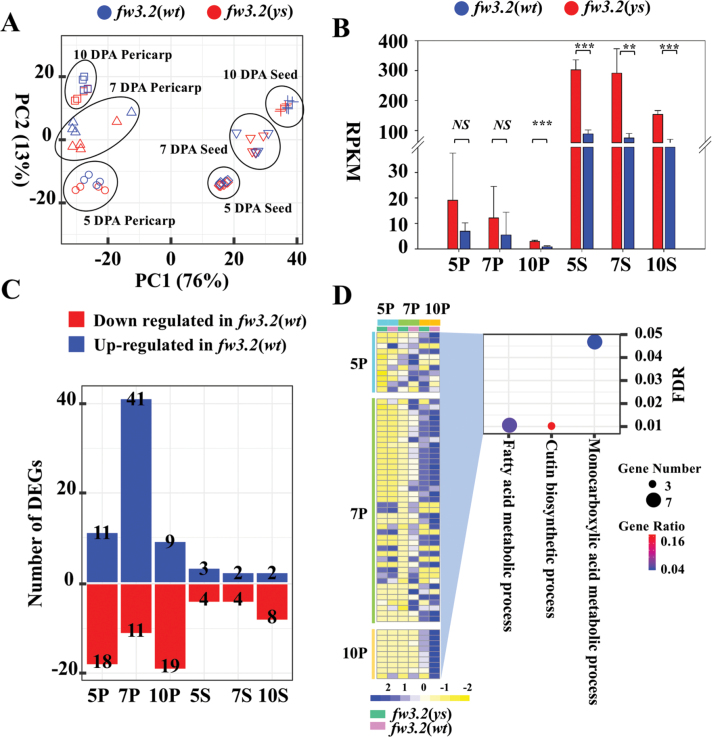
Differential gene expression analyses in developing pericarp and seed between the *fw3.2* NILs. (A) PCA plot showing the clustering of transcriptomes from pericarp and seed tissues at different time points in the *fw3.2* NILs. Each data point represents a biological replicate. (B) Expression of *SlKLUH* in pericarp and seed tissues at different time points in the *fw3.2* NILs. Asterisks denote significant differences (**P<0.01; ***P<0.001) as determined by Student’s *t*-tests. NS, non-significant difference. (C) DEGs at different developmental time points of pericarp and seed. (D) Different expression patterns (left panel) and GO enrichment (right panel) of up-regulated DEGs in the pericarp of *fw3.2*(*wt*). The size of the circles indicates the number of DEGs in the given GO term. The color coding indicates the gene ratio calculated as the number of DEGs in the given GO term divided by the total number of genes in the term. The numbers 5, 7, and 10 indicate 5 DPA, 7 DPA, and 10 DPA, respectively. P, pericarp; S, seed.

To gain further insights into the molecular mechanisms of SlKLUH governing fruit and seed weight in tomato, a gene expression analysis was performed using RNA isolated from the NIL tissues corresponding to developing pericarp and seed at 5, 7, and 10 DPA. The SCC analysis showed high reproducibility between the four replicates, ranging from 0.97 to 0.98 (Supplementary Fig. S3). Moreover, the PCA showed that the samples clustered based on tissue type and developmental time point but less based on genotype (Fig. 3A). This suggests that the overall transcriptome profiles did not differ dramatically between the NILs.

RNA expression analyses showed that *SlKLUH* (*Solyc03g114940*) was significantly more highly expressed in *fw3.2*(*ys*) than in *fw3.2*(*wt*) in most of the samples analyzed (Fig. 3B). DEGs between the NILs were identified by six pairwise comparisons of pericarp and seed at each developmental time point (Fig. 3C; Supplementary Dataset S1). In the small-fruited NIL *fw3.2*(*wt*), 48 unique DEGs exhibited significantly lower expression and 61 unique DEGs exhibited significantly higher expression at different time points in the developing pericarp and seed compared with the large-fruited NIL *fw3.2*(*ys*). Notably, fewer DEGs were found in seed (16 unique genes) than in the pericarp (97 unique genes) (Fig. 3C; Supplementary Dataset S1), demonstrating more changes in gene expression during the development of the pericarp than during development of the seed as a consequence of differential expression of *SlKLUH*. This was despite the fact that *SlKLUH* itself was much more highly expressed in the seed (Fig. 3B). GO enrichment analysis of up-regulated DEGs in the pericarp of *fw3.2*(*wt*) indicated that the DEGs are enriched for three processes related to lipid metabolism, namely ‘Fatty acid metabolic process’ (six genes), ‘Cutin biosynthetic process’ (three genes), and ‘Monocarboxylic acid metabolic process’ (seven genes) (Fig. 3D). However, no significantly enriched biological processes were identified for the down-regulated DEGs in pericarp and in seed of *fw3.2*(*wt*). Consequently, this finding implied that lower expression of *SlKLUH* led to up-regulation of lipid metabolism-related processes.

To systematically explore the RNA-seq data, linear factorial modeling was applied to identify DEGs significantly affected by genotype, genotype by tissue interaction (G×T), and genotype by developmental stage interaction (G×D). A total of 72 DEGs, which were consistently up- or down-regulated in *fw3.2*(*wt*) across all samples, were identified with significant genotype effects (Supplementary Dataset S2). As expected, *SlKLUH* was a DEG significantly affected by genotype, with lower expression in pericarp and seed at all developmental stages in *fw3.2*(*wt*) compared with *fw3.2*(*ys*) (Fig. 3B; Supplementary Dataset S2). No DEGs were found with significant G×T and G×D effects.

### RNA-seq analysis of the RNAi lines of *SlKLUH*

**Fig. 4.**
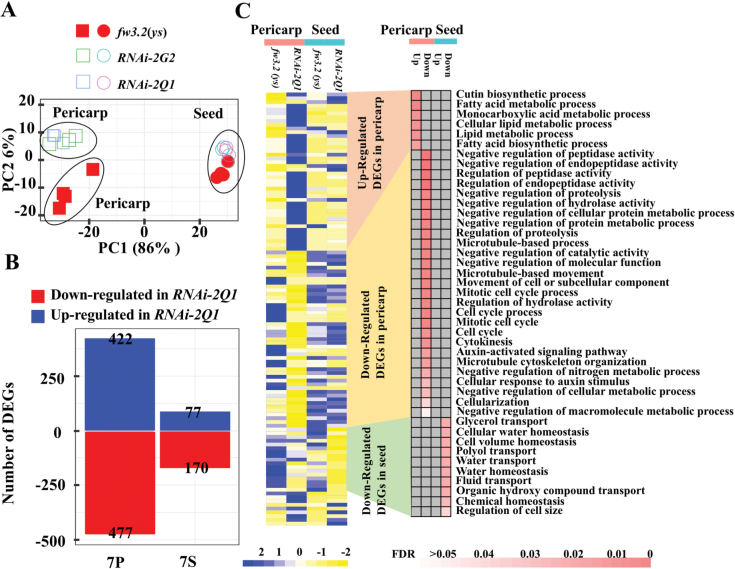
Transgenic down-regulation of *SlKLUH* affects the transcriptome of pericarp and seed at 7 DPA. (A) PCA plot showing the clustering of transcriptomes from 7 DPA pericarp and seed in *fw3.2*(*ys*) and the *RNAi-2G2* and *RNAi-2Q1* lines that down-regulate the expression of *SlKLUH*. Each data point represents a biological replicate. (B) DEGs in 7 DPA pericarp and seed in the *fw3.2*(*ys*)–*RNAi-2Q1* comparison. 7, 7 DPA; P, pericarp; S, seed; (C) Heatmap showing different expression patterns (left panel) and GO enrichment (right panel) of DEGs in the *fw3.2*(*ys*)–*RNAi-2Q1* comparison. The gray boxes in the right-hand panel represent missing GO terms. Only the enriched GO terms with adjusted *P*-value <0.05 are shown.

Even though the natural *fw3.2* NILs show changes in *SlKLUH* expression, further down-regulation of the gene may lead to the identification of additional DEGs in the SlKLUH pathway. *RNAi-2Q1* and *RNAi-2G2* were two independent transgenic lines that down-regulated the expression of *SlKLUH* in the *fw3.2*(*ys*) background. These plants showed significantly reduced fruit and seed weight compared with *fw3.2*(*ys*) and *fw3.2*(*wt*) (Chakrabarti *et al.*, 2013). RNA-seq was performed using total RNA isolated from the 7 DPA pericarp and seed of the *RNAi-2Q1* and *RNAi-2G2* lines. The correlation coefficient between the three biological replicates of different tissues varied from 0.97 to 0.98, indicating the high correlation among the samples (Supplementary Fig. S4). A PCA was performed to obtain a general view of the transcriptome changes between the *RNAi-2Q1* and *RNAi-2G2* lines and their control *fw3.2*(*ys*). The analysis revealed that PC1 separated pericarp tissues from seed tissues, explaining 86% of the variance. PC2 separated the tissues based on genotype, explained 6% of the variance, and showed clear clustering in the pericarp based on genotype (Fig. 4A).

Given that the overall transcriptome profiles of 7 DPA pericarp and seed were similar between *RNAi-2Q1* and *RNAi-2G2* (Fig. 4A; Supplementary Fig. S4), we focused on *RNAi-2Q1* expression data for further analysis. A total of 899 and 247 DEGs were identified in 7 DPA pericarp and seed of the *RNAi-2Q1* line, respectively (Fig. 4B; Supplementary Dataset S3). As expected, the total number of DEGs in the *fw3.2*(*ys*)–*RNAi-2Q1* dataset was much higher than that of the *fw3.2*(*ys*)–*fw3.2*(*wt*) dataset (Figs 3C, 4B), possibly resulting from the more extensive down-regulation of *SlKLUH* by RNAi compared with the NILs (Chakrabarti *et al.*, 2013). In 7 DPA pericarp, six down-regulated and 33 up-regulated genes were shared in both datasets (Supplementary Fig. S5; Supplementary Table S2). Interestingly, *SlKLUH* was the only common DEG that was down-regulated in both RNA-seq datasets in 7 DPA seed (Supplementary Fig. S5; Supplementary Table S2).

Similar to the GO term enrichment of the up-regulated DEGs in pericarp of the *fw3.2*(*wt*), the DEGs that were up-regulated in *RNAi-2Q1* pericarp were also enriched for ‘Fatty acid metabolic process’, ‘Cutin biosynthetic process’, and ‘Monocarboxylic acid metabolic process’ (Figs 3D, 4C). Again, the reduced expression of *SlKLUH* led to enhanced expression of lipid metabolism-related genes. The enriched GO terms of the down-regulated genes in 7 DPA pericarp of *RNAi-2Q1* included terms related to cellular processes, such as ‘Microtubule-based process’, ‘Cell cycle process’, and ‘Microtubule cytoskeleton organization’. Genes involved in these processes included putative orthologs of the Arabidopsis *ATAURORA1* (*AUR1*) (*Solyc08g066050*), *ARABIDOPSIS NPK1-ACTIVATING KINESIN 1* (*ATNACK1*) (*Solyc03g119220*), *MICROTUBULE-ASSOCIATED PROTEIN 65-3* (*MAP65-3*) (*Solyc03g007130*), and *TETRASPORE* (*Solyc07g042560*). In addition, down-regulated DEGs in the *RNAi-2Q1* developing seeds were primarily associated with processes related to transport and homeostasis, whereas up-regulated DEGs in the seeds were not enriched for any biological processes (Fig. 4C). Collectively, the GO enrichment analyses of DEGs from both expression studies suggested that decreased expression of *SlKLUH* in *fw3.2*(*wt*) and the *RNAi-2Q1* results in smaller sizes of fruit and seed possibly by increasing lipid metabolism.

### Characterization of DEGs involved in lipid metabolism pathways

**Fig. 5.**
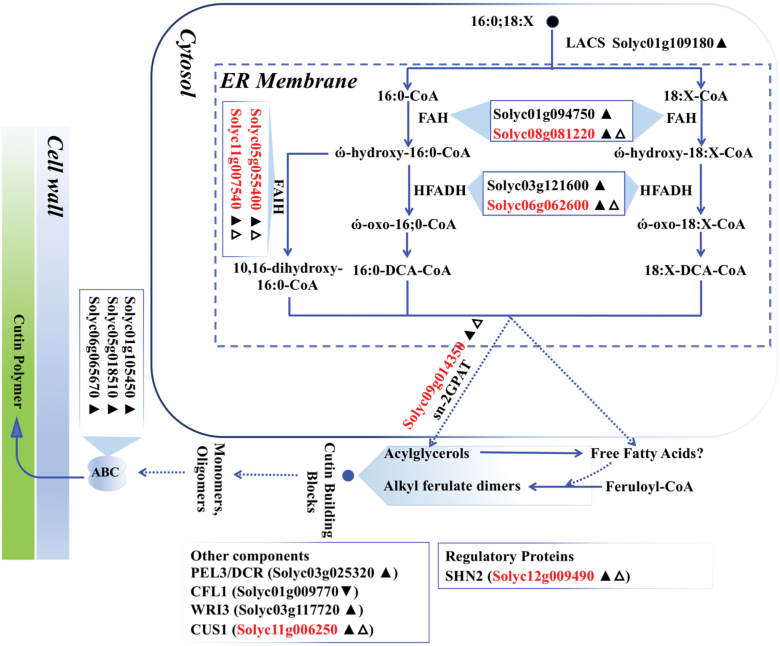
*SlKLUH* expression is associated with genes involved in ‘Cutin synthesis and transport’ pathway. Schematic overview of the ‘Cutin synthesis and transport’ pathway was modified from the ARABIDOPSIS ACYL-LIPID METABOLISM database (http://aralip.plantbiology.msu.edu/pathways/pathways). For additional details on genes involved in this pathway, see http://aralip.plantbiology.msu.edu/pathways/cutin_synthesis_transport and http://aralip.plantbiology.msu.edu/pathways/cutin_synthesis_transport_2. The filled upright and inverted triangles indicate up- and down-regulated DEGs in the *RNAi-2Q1* line compared with *fw3.2*(*ys*), respectively. The open upright triangle indicates up-regulated DEGs in *fw3.2*(*wt*) compared with *fw3.2*(*ys*); red gene ID indicates the DEGs shared between the two DEG datasets.

**Fig. 6.**
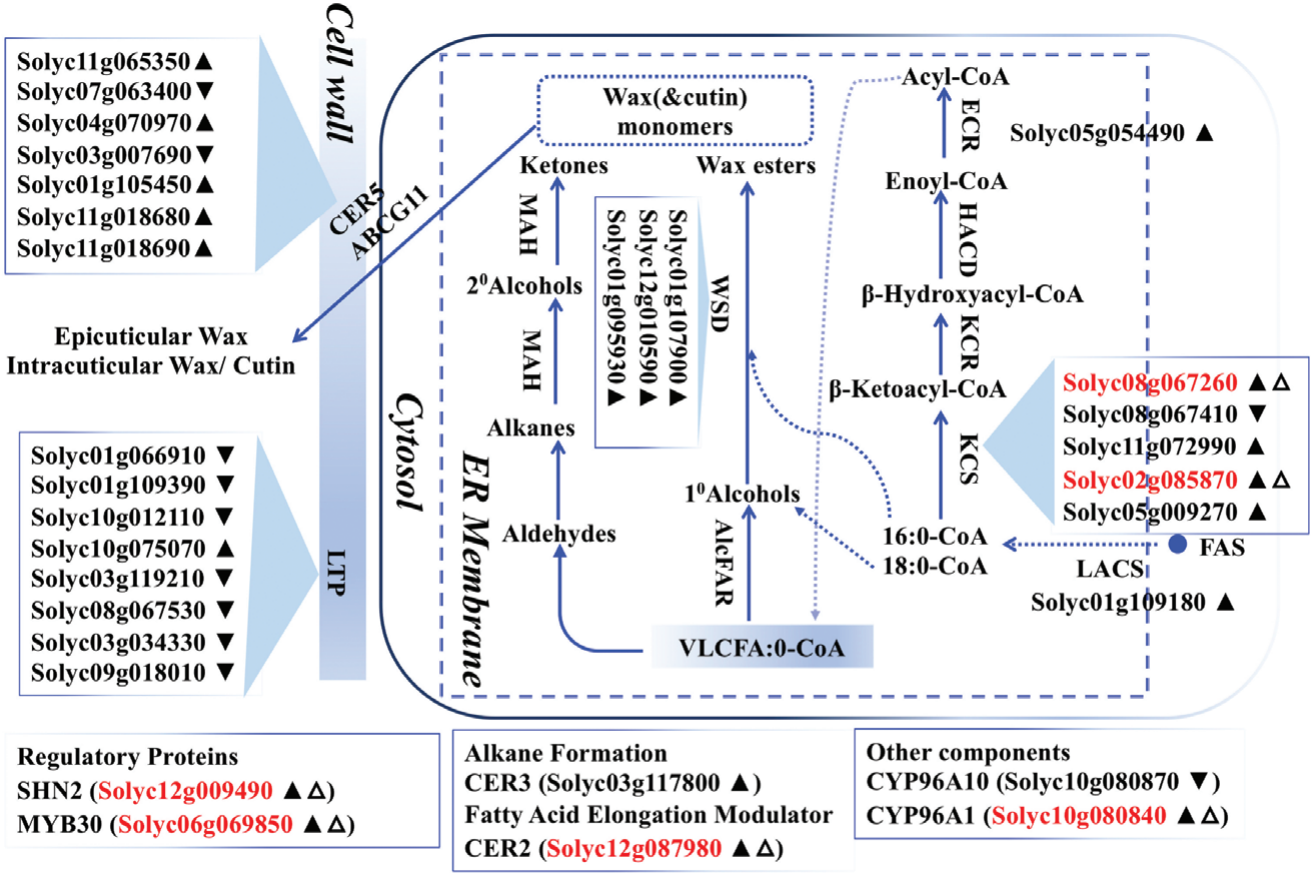
*SlKLUH* impacts genes involved in ‘Fatty acid elongation and wax biosynthesis’ pathway. Schematic overview of the ‘Fatty acid elongation and wax biosynthesis’ pathway was modified from the ARABIDOPSIS ACYL-LIPID METABOLISM database (http://aralip.plantbiology.msu.edu/pathways/pathways). For additional details on genes involved in this pathway, see http://aralip.plantbiology.msu.edu/pathways/fatty_acid_elongation_wax_biosynthesis. The filled upright and inverted triangles indicate up- and down-regulated DEGs in the *RNAi-2Q1* line compared with *fw3.2*(*ys*), respectively. The open upright triangle indicates up-regulated DEGs in *fw3.2*(*wt*) compared with *fw3.2(ys)*; red gene ID indicates the DEGs shared between the two DEG datasets.

The DEGs were mapped onto pathways using the ACYL-LIPID METABOLISM database (http://aralip.plantbiology.msu.edu/pathways/pathways). We detected 23 and 101 lipid metabolism-related DEGs in the *fw3.2*(*ys*)–*fw3.2*(*wt*) dataset and in the *fw3.2*(*ys*)–*RNAi-2Q1* dataset (Supplementary Table S3), respectively. ‘Cutin synthesis and transport’ and ‘Fatty acid elongation and wax biosynthesis’ were the two most abundant lipid metabolism pathways shared by the *fw3.2*(*ys*)–*fw3.2*(*wt*) and the *fw3.2*(*ys*)–*RNAi-2Q1* datasets (Supplementary Fig. S6). We found 16 DEGs (seven DEGs were shared between the two datasets) and 31 DEGs (six DEGs were shared between the two datasets) involved primarily in ‘Cutin synthesis and transport’ (Fig. 5) and ‘Fatty acid elongation and wax biosynthesis’ (Fig. 6), respectively. Importantly, most of the DEGs (15 out of 16 DEGs involved in ‘Cutin synthesis and transport’ and 20 out of 31 DEGs involved in ‘Fatty acid elongation and wax biosynthesis’) were more highly expressed in *fw3.2*(*wt*) and/or the *RNAi-2Q1* line than in *fw3.2*(*ys*). The increased expression of the lipid-related genes involved in the two pathways and the concomitant decreased fruit and seed weight in the *fw3.2*(*wt*) and the *RNAi-2Q1* lines suggested a functional correlation between lipid metabolism and fruit/seed size regulation.

### Differential accumulation of lipids

**Fig. 7.**
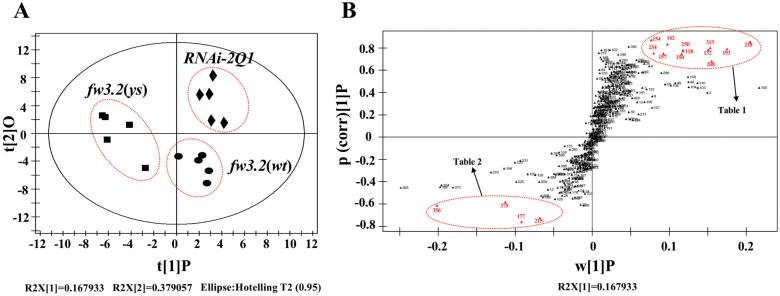
OPLS-DA of lipidome data of 5 DPA fruit from *fw3.2*(*ys*), *fw3.2*(*wt*), and *RNAi-2Q1*. (A) Score plot (R2X[1]=0.167933, R2X[2]=0.379057). The samples from *fw3.2*(*ys*), *fw3.2*(*wt*), and *RNAi-2Q1* are clearly separated. Each dot represents an individual sample. (B) S-plot of OPLS-DA based on ANOVA of the cross-validated residuals (CV-ANOVA). Each point represents a lipid molecule. The variables that did not significantly vary are plotted in the middle. The lipids that changed most contributed to the class separation and are plotted at the top or bottom of the S-shaped plot in red. The discriminative metabolites whose levels found in *fw3.2*(*wt*) and *RNAi-2Q1* were higher than those from *fw3.2*(*ys*) are shown in the upper right region of the S-plot, while the discriminative metabolites whose levels from *fw3.2*(*wt*) and *RNAi-2Q1* were lower than those of *fw3.2*(*ys*) are shown in the lower left region. Details of discriminative metabolites in the upper right and lower left are shown in Tables 1 and 2.

The gene expression data implied that changes in lipid metabolism were a consequence of the differential expression of *SlKLUH*. To further investigate the effects of *SlKLUH* on lipid metabolism, we performed lipid profiling of 5 DPA fruits from the NILs and *RNAi-2Q1*. A total of 425 metabolites were detected and 58 were annotated as signals derived from known lipids (Supplementary Fig. S7). An OPLS-DA (Wiklund *et al.*, 2008) was performed to identify the major difference in the lipid profile between the different genotypes. The OPLS-DA (Fig. 7A) and OPLS loading S-plot (Fig. 7B) revealed that the discriminative metabolites with significantly increased levels in *fw3.2*(*wt*) and *RNAi-2Q1* were non-phosphorus glycerolipids, including monogalactosyldiacylglycerol (MGDG) and digalactosyldiacylglycerol (DGDG), and phospholipids, including phosphatidylcholine (PC), phosphatidylethanolamine (PE), phosphatidylinositol (PI), and four unknown lipids (Table 1). Only four unknown lipids were significantly decreased in *fw3.2*(*wt*) and the *RNAi-2Q1* line (Table 2). This result provides information about the effects of *SlKLUH* expression levels on lipid metabolites. However, details of the biosynthesis of the discriminative lipids and the mechanisms by which SlKLUH affects their accumulation remain unknown.

**Table 1.** Discriminative metabolites predicted by OPLS-DA with increased levels in *fw3.2*(*wt*) and *RNAi-2Q1*

Retention time (min)	*m/z*	Annotation	Averaged intensity (mean ±SD)		
			*fw3.2*(*ys*)	*fw3.2*(*wt*)	*RNAi-2Q1*
4.49	716.522	PE_34:1 ([M-H]^–^)	0.40±0.092	0.48±0.068	0.57±0.067
4.31	740.522	PE_36:3 ([M-H]^–^)	0.75±0.13	1.1±0.14	1.25±0.29
4.39	745.556	Unknown	0.60±0.059	0.72±0.060	0.71±0.11
4.40	744.553	Unknown	1.3±0.11	1.6±0.078	1.6±0.26
4.20	768.553	Unknown	0.82±0.12	1.1±0.10	1.3±0.29
4.40	804.575	PC_34:1 ([M+HCOO]^–^)	4.8±0.50	5.6±0.21	5.7±0.92
3.82	819.526	MGDG_36:6 ([M+HCOO]^–^)	21±1.7	24±2.2	26±1.5
4.20	828.575	PC_36:3 ([M+HCOO]^–^)	3.4±0.33	4.3±0.42	5.3±0.99
3.99	835.533	PI_34:1 ([M-H]^–^)	1.3±0.16	1.6±0.099	1.5±0.22
4.81	832.606	PC_36:1 ([M+HCOO]^–^)	0.85±0.14	0.93±0.021	0.93±0.20
3.56	935.574	Unknown	13±0.71	14±0.86	14±0.56
3.56	981.579	DGDG_36:6 ([M+HCOO]^–^)	9.7±0.49	10±0.69	10±0.37

DGDG, digalactosyldiacylglycerol; MGDG, monogalactosyldiacylglycerol; SQDG, sulfoquinovosyldiacylglycerol; PC, phosphatidylcholine; PE, phosphatidylethanolamine; PI, phosphatidylinositol.

**Table 2.** Discriminative metabolites predicted by OPLS-DA with decreased levels in *fw3.2*(*wt*) and *RNAi-2Q1*

Retention time (min)	*m/z*	Annotation	Averaged intensity (mean ±SD)		
			*fw3.2*(*ys*)	*fw3.2*(*wt*)	*RNAi-2Q1*
4.17	786.528	Unknown	0.58±0.028	0.56±0.10	0.45±0.07
4.57	814.559	Unknown	1.1±0.094	1.0±0.12	0.9±0.11
0.27	1068.510	Unknown	5.4±0.80	5.5±1.0	5.1±0.58
0.31	1126.515	Unknown	0.74±0.26	0.79±0.33	1.2±0.25

### Identification of gene co-expression modules in *fw3.2*(*ys*) and *fw3.2*(*wt*) by WGCNA

**Fig. 8.**
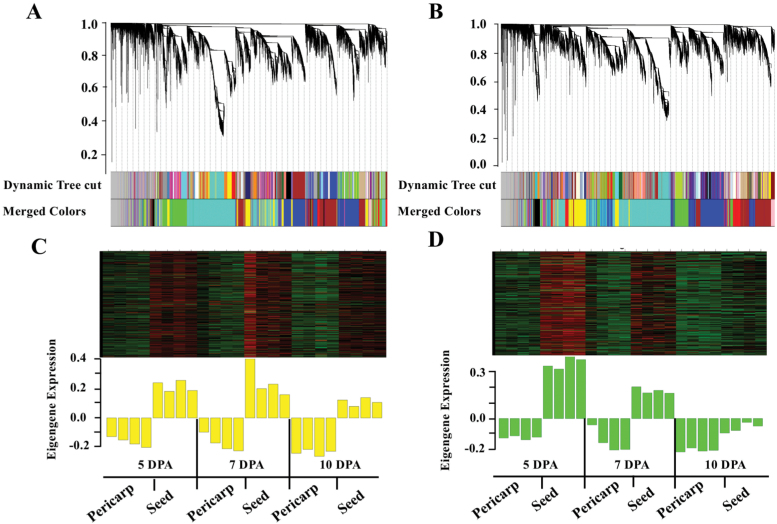
Co-expression analyses with *SlKLUH* in developing pericarp and seed in the *fw3.2* NILs. (A) Hierarchical cluster tree of genes showing co-expression modules based on WGCNA in *fw3.2*(*ys*). (B) Hierarchical cluster tree of genes showing co-expression modules based on WGCNA in *fw3.2*(*wt*). Each ‘leaf’ in the tree represents one individual gene. The branches correspond to modules labeled with different colors. The color rows below the dendrograms indicate module membership in *fw3.2*(*ys*) (A) and in *fw3.2*(*wt*) (B). (C) Heatmap of gene expression (upper panel) and expression levels of the corresponding eigengene across the samples (lower panel) in the *fw3.2*(*ys*) NIL. The heatmap (upper panel) and barplot of eigengene expression (lower panel) have the same samples (*x*-axis). Rows of the heatmap correspond to genes, columns to samples. Red and green in the color key denote overexpression and underexpression, respectively. (D) Similar to (C) but instead for *fw3.2*(*wt*).

To identify pathways that are consistently associated with *SlKLUH* expression, WGCNA was performed using the RNA-seq data from either *fw3.2*(*ys*) or *fw3.2*(*wt*). The networks identified from this analysis might be directly linked to the function of SlKLUH in regulating organ size. A total of 11 modules (comprised of 43–4199 genes) were identified in *fw3.2*(*ys*) (Fig. 8A; Supplementary Dataset S4), and 10 modules (comprised of 48–4845 genes) were recognized in *fw3.2*(*wt*) (Fig. 8B; Supplementary Dataset S5). *SlKLUH* was assigned to the yellow module (YYM) in *fw3.2*(*ys*) containing 1676 genes (Supplementary Dataset S4), whereas this gene was assigned to the green module (WGM) in *fw3.2*(*wt*) containing 1245 genes (Supplementary Dataset S5). The eigengenes of *fw3.2*(*ys*) YYM (Fig. 8C) and *fw3.2*(*wt*) WGM (Fig. 8D) showed consistently higher expression in seeds compared with pericarp, which mirrored the expression levels of *SlKLUH*.

**Fig. 9.**
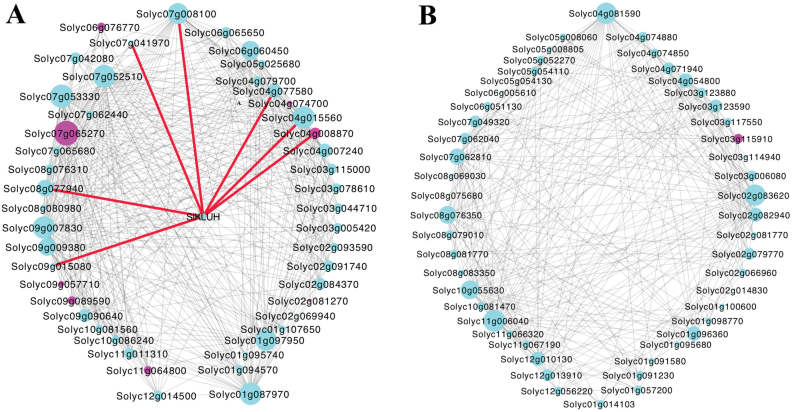
Network depiction of the *SlKLUH*-containing modules with hub genes. (A) YYM network in *fw3.2*(*ys*); (B) WGM network in *fw3.2*(*wt*). Fifty hub genes with the edge weight higher than 0.25 (A) and 0.2 (B), respectively, are visualized by Cytoscape. The pink circles represent TFs. Red lines show the edges of *SlKLUH* to its neighbor genes. Nodes represent genes, and node size is correlated with connectivity of the gene.

Of the 1676 genes in YYM, 421 (~25.1%) were shared with the co-expressed genes in WGM (Supplementary Fig. S8). This relatively low overlap suggested that the module-specific transcriptome profile was noticeably changed as a result of higher *SlKLUH* expression. Genes with the highest *k*_ME_ values are referred to as intramodular hub genes and are thought to play critical roles in maintaining network structure and function (Barabási *et al.*, 2011; Langfelder *et al.*, 2013). We found that *SlKLUH* (*k*_ME_=0.971) ranked 32nd and formed a hub gene in YYM (Fig. 9A; Supplementary Dataset S4). This suggested that *SlKLUH* acted to maintain the network structure and function for this module. In contrast, *SlKLUH* (*k*_ME_=0.885) in the *fw3.2*(*wt*) dataset ranked 366th, and was therefore not a hub gene (Fig. 9B; Supplementary Dataset S5).

**Fig. 10.**
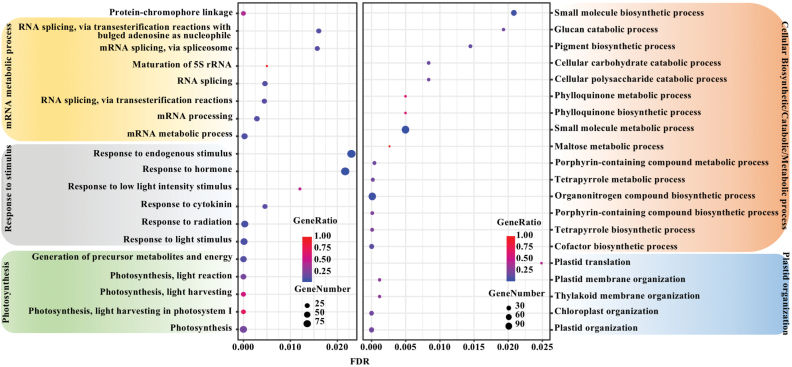
Significantly enriched GO terms of the YYM (left panel) and WGM (right panel). The size of the circles indicates the number of co-expressed genes in the given GO term. The color coding indicates the gene ratio calculated as the number of co-expressed genes in the given GO term divided by the total number of genes in the term. The *x*-axis indicates the FDR-adjusted *P*-value.

GO term enrichment analysis identified genes in YYM related to ‘Photosynthesis’, ‘Response to stimulus’, and ‘mRNA metabolic process’ (Fig. 10). The genes in WGM were enriched for ‘Plastid organization’ and ‘Cellular metabolic/catabolic/biosynthetic process’ (Fig. 10). Even though no common enriched GO terms were identified, many enriched GO term categories relate to photosynthesis, chloroplast organization, and chlorophyll biosynthesis. These results suggested a common theme of *SlKLUH*-co-expressed genes in *fw3.2*(*ys*) and *fw3.2*(*wt*) that impact chloroplast functioning such as in carbon fixation which might possibly be directly related to organ growth. In addition, no GO terms were enriched in the shared set of 421 genes.

### Differentially and co-expressed transcription factors are implicated in tomato fruit and seed weight control mediated by *SlKLUH*

Gene expression dynamics of those involved in lipid metabolism and photosynthesis-related processes are regulated directly by TFs. In the DEG analyses, nine TFs were shared in the *fw3.2*(*ys*)–*fw3.2*(*wt*) and *fw3.2*(*ys*)–*RNAi-2Q1* datasets (Supplementary Table S4), suggesting that these TFs might play important roles in regulating fruit and seed weight in the KLUH pathway. For example, *Solyc04g074990* (ZF-HD), *Solyc08g079800* (GRF), and *Solyc04g077510* (GRF) were down-regulated in 7 DPA pericarp in both *fw3.2*(*wt*) and the *RNAi-2Q1* line (Supplementary Figs S5, S9; Supplementary Datasets S1, S3; Supplementary Table S4). GRFs are known as positive regulators of primary cell proliferation and play important roles in regulating organ size in plants (Horiguchi *et al.*, 2005; Vercruyssen *et al.*, 2015; Li *et al.*, 2016; Shimano *et al.*, 2018; Zhang *et al.*, 2018). *Solyc04g074990* encodes a ZF-HD TF that is closely related to Arabidopsis *HB22*, *HB25*, and *HB33*. In Arabidopsis, overexpression of *ATHB25* results in wider siliques and larger seeds, while simultaneous knockdown of *ATHB25*, *ATHB22*, and *ATHB31* leads to smaller seeds (Bueso *et al.*, 2014). Therefore, the down-regulation of *Solyc04g074990*, *Solyc08g079800*, and *Solyc04g077510* was associated with smaller fruit and seed in *fw3.2*(*wt*) and *RNAi-2Q1*, suggesting that they may function as positive regulators of tomato fruit and seed size. However, further study is required to dissect their exact roles in fruit and seed weight regulation mediated by KLUH in tomato.

The DEGs in the TF category are considered to change expression as an indirect consequence of the expression level of *SlKLUH*. However, these DEGs may or may not be found in the same module as *SlKLUH*. TF genes that are found in the same module as *SlKLUH* may be more directly involved in the entire KLUH network. To obtain further insight into the transcriptional regulation of the KLUH pathway, we sought out the TFs in these two modules. The YYM harbored 117 TFs (6.98%) which were classified into 35 families. The 10 most abundant TF families in YYM were bHLH (13), C2H2 (11), MYB (9), MYB-related (6), B3 (6), Trihelix (5), bZIP (5), ERF (5), AP2 (4), and NAC (4) (Supplementary Fig. S10A; Supplementary Table S4). The WGM contained 76 TFs (6.10%) mainly from families classified as bHLH (12), C2H2 (6), HD-ZIP (4), GRAS (4), MYB (3), MYB-related (3), NAC (3), Trihelix (3), bZIP (3), and Dof (3) (Supplementary Fig. S10B; Supplementary Table S4). Thirty-seven TFs were shared by YYM and WGM (Supplementary Table S4). The orthologs of some of common TFs were known from other studies to participate in organ size regulation in plants. For example, *VAL1* (*AT2G30470*) (*Solyc06g082520*), a member of the B3 domain TFs and a negative regulator of oil production, plays a major role in plant embryo development (Tsukagoshi *et al.*, 2005, 2007; Suzuki *et al.*, 2007; Schneider *et al.*, 2016). The putative ortholog of Arabidopsis *SUPERMAN* (*SUP*; *AT3G23130*), *Solyc09g089590*, encodes a zinc-finger protein that in Arabidopsis has been proposed to control cell proliferation by regulating the transcription of genes that affect cell division, thus regulating organ size (Hiratsu *et al.*, 2002; Nibau *et al*., 2010). Interestingly, three of the 37 TF geness (*Solyc02g089540*, *Solyc06g060830*, and *Solyc11g072470*) were also identified as DEGs in the *fw3.2*(*ys*)–*RNAi-2Q1* dataset (Supplementary Table S4). The putative tomato *HB2* (*Solyc06g060830*) was up-regulated in pericarp of *RNAi-2Q1*. In Arabidopsis, overexpression of *AtHB2* (*AT4G16780*) significantly affects the α-linolenic acid and total fatty acid contents as well as plant growth and seed dry weight (Vigeolas *et al.*, 2011; Nehlin, 2015; Ivarson *et al*., 2017). One of the *LATERAL ORGAN BOUNDARIES DOMAIN* (*LBD*) family of TF genes, *Solyc11g072470*, was also up-regulated in the pericarp of *RNAi-2Q1*. *Populus LBD1* is involved in the regulation of secondary growth in *Populus* and an activation-tagged mutant of *PtaLBD1* showed increased stem diameter and smaller leaves (Yordanov *et al.*, 2010).

Other TF genes previously described as organ size regulators were co-expressed with *SlKLUH* in either YYM or WGM. For example, four *AP2* TF genes were identified in YYM but not in WGM (Supplementary Figs S10, S11; Supplementary Table S4). Of these, three *AP2* TF genes (*Solyc02g064960*, *Solyc10g084340*, and *Solyc04g049800*) were clustered with the APETALA-like subfamily (Supplementary Fig. S11). *AT4G36920*, a member of the APETALA-like subfamily, plays an important role in determining seed size and oil contents without substantial changes in seed fatty acid composition (Jofuku *et al.*, 2005; Ohto *et al.*, 2009; Yan *et al.*, 2012). In rice, *SUPERNUMERARY BRACT* (*OsSNB*) was identified as a negative regulator of seed weight (Jiang *et al.*, 2019). In tomato, *AP2a* (*Solyc03g044300*) was identified as a major negative regulator of fruit ripening via regulation of ethylene biosynthesis and signaling (Chung *et al.*, 2010; Karlova *et al.*, 2011). The RNAi lines of *SlAP2a* showed smaller fruit size than the wild type (Chung *et al.*, 2010). Another *AP2* TF gene, *Solyc02g030210*, is one of the orthologs of *WRINKLED1* (*WRI1*) (Supplementary Fig. S11). The positive roles of AtWRI1 and its orthologs in regulating lipid metabolism and seed mass have been extensively demonstrated (Baud *et al.*, 2009; Shen *et al.*, 2010; Qu *et al.*, 2012; Ma *et al.*, 2013; Wu *et al.*, 2014; Ivarson *et al*., 2017). In addition, putative auxin response factor (ARF) genes (*Solyc07g043620*, *Solyc07g043610*, and *Solyc07g016180*) and *WUSCHEL-related homeobox* (*WOX*) (*Solyc02g077390*) were identified as co-expressed genes of *SlKLUH* in WGM only (Supplementary Table S4). Many ARFs regulate gene expression in response to auxin, and have been identified as important regulators of organ size, including ARF2 in Arabidopsis (Schruff *et al.*, 2006), ARF1 in rice (Aya *et al.*, 2014), ARF18 in rapeseed (Liu *et al.*, 2015), ARF2 in sea buckthorn (Ding *et al.*, 2018), and ARF19 in the woody plant *Jatropha curcas* (Y. Sun *et al.*, 2017). In addition, several ARFs in cucumber were identified which putatively regulate carpel number variation through interaction with the orthologs of CLV3 and WUS (Che *et al.*, 2020). WOXs are also well known to be associated with organ size. For example, overexpression of *STENOFOLIA* (*STF*), a WOX family TF gene, significantly increases plant size, including leaf width and stem thickness, through enhancing cell proliferation (Wang *et al.*, 2017). These data suggest that TFs may play a key role in fruit and seed weight regulation in the KLUH pathway in tomato.

### Overexpression of *SlSHN1* significantly decreases fruit and seed weight

**Fig. 11.**
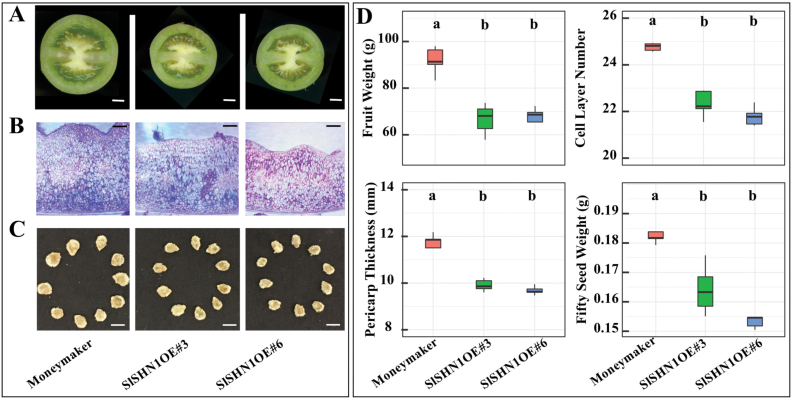
Overexpression of *SlSHN1* results in decreased fruit and seed weight. (A) Medio-lateral section of mature fruits of cultivar Moneymaker and *SlSHN1* overexpression lines in the Moneymaker background. Scale bar=1 cm. (B) Hand cut section of pericarp from representative mature green fruit stained with toluidine blue. Scale bar=2 mm. (C) Representative seeds from Moneymaker and the *SlSHN1* overexpression lines. Scale bar=2 mm. (D) Quantitative analysis of fruit weight, pericarp thickness, cell layer number, and seed weight. For pericarp cell layer analysis, the endoderm layer and the small cells below the exoderm were not counted since in many cases they are not clearly visible which could skew the results. The letters in the boxplots indicate significant differences among different genotypes evaluated by Duncan’s test (α<0.05).

In tomato, certain lipid metabolism-related genes have been experimentally characterized for their involvement in fruit cutin biosynthesis and fatty acid elongation. To evaluate if genes that impact these two pathways also play roles in fruit and seed weight, we identified the sources of transgenic or natural mutant lines that differ in their lipid metabolism. One example is WAX INDUCER1/SHINE1 (WIN/SHN1) which encodes a transcription factor that regulates the ‘Cutin synthesis and transport’ and ‘Fatty acid elongation and wax biosynthesis’ pathways in Arabidopsis (Aharoni *et al.*, 2004; Broun *et al.*, 2004; Kannangara *et al.*, 2007; Li-Beisson *et al*., 2010, 2013). In tomato, *SHINE1* (*SlSHN1*; *Solyc03g116610*) and *SlSHN2* (*Solyc12g009490*) are putative orthologs of Arabidopsis *WIN*/*SHN1*. *SlSHN2* showed significantly higher expression in the pericarp of *fw3.2*(*wt*) (Supplementary Dataset S2) and the *RNAi-2Q1* line (Supplementary Dataset S3) compared with *fw3.2*(*ys*). On the other hand, *SlSHN1* showed low or undetectable expression in our datasets (0–0.1 RPKM). Overexpression of *SlSHN1* increases cuticular wax accumulation, resulting in improved drought tolerance in tomato (Al-Abdallat *et al.*, 2014). To assess whether overexpression of *SlSHN1* affects fruit and seed weight, we evaluated these traits in the previously described *SlSHN1*-overexpressing lines (Al-Abdallat *et al.*, 2014). The results showed that high and ubiquitous expression of *SlSHN1* significantly decreased fruit weight by reducing the number of cell layers and pericarp thickness compared with the non-transgenic control (Fig. 11). Seed weight was also reduced in the overexpression lines (Fig. 11). The results imply that fruit and seed weight may be directly affected by changes in lipid metabolism by the paralog of *SlSHN1*, *SlSHN2*.

Tomato *CD2* (*Solyc01g091630*), a HD-Zip TF gene, was found in the WGM, and is involved in cutin biosynthesis and wax deposition (Nadakuduti *et al.*, 2012; Kimbara *et al.*, 2013). We evaluated the fruit and seed weight of the *cd2* mutant in the AC background. No significant differences in fruit and seed w eight were found between *cd2* and the control under greenhouse conditions (Supplementary Fig. S12A). In the field trial, fruit weight in *cd2* was significantly higher than in the control, whereas seed weight was significantly lower than in the control (Supplementary Fig. S12B). The inconsistent results between the greenhouse and field trials as well as between the fruit and seed suggested that these traits were not significantly affected by *CD2*.

## Discussion

### SlKLUH appears to function in the cell proliferation phase at the early stages of fruit development

CYP78A is a highly conserved plant-specific subclade in the CYP450 family (Nelson, 2006; Mizutani and Ohta, 2010). Members of CYP78A are recognized to positively regulate organ weight and size as well as development in plants such as Arabidopsis (Wang *et al.*, 2008; Fang *et al.*, 2012; Sotelo-Silveira *et al.*, 2013; Yang *et al.*, 2013), rice (Nagasawa *et al.*, 2013; Yang *et al.*, 2013; Maeda *et al.*, 2019), wheat (Ma *et al*., 2015*a*, *b*), maize (X. Sun *et al.*, 2017), soybean (Wang *et al.*, 2015*a*; Zhao *et al.*, 2016), *J. curcas* (Tian *et al.*, 2016), sweet cherry (Qi *et al.*, 2017), tomato, and pepper (Chakrabarti *et al.*, 2013). Different CYP78As regulate organ size differently. For example, Arabidopsis KLUH/CYP78A5 appears to affect cell proliferation at the early stages of integument growth, therefore regulating the seed size (Anastasiou *et al.*, 2007; Adamski *et al.*, 2009). In contrast, EOD3/CYP78A6 and CYP78A9 are primarily involved in the regulation of cell expansion phases during the later stages of integument development (Fang *et al.*, 2012). Moreover, the rice and maize *PLASTOCHRON1* (*PLA1*) genes stimulate leaf growth by prolonging the duration of cell division (Miyoshi *et al.*, 2004; Mimura and Itoh, 2014; X. Sun *et al.*, 2017). In this study, we found that SlKLUH affects pericarp cell proliferation in the early stages of fruit development (5–7 DPA). The phylogenetic analysis supported the notion that SlKLUH controls cell proliferation as it is the closest ortholog to AtCYP78A10 and AtCYP78A5 (Fig. 1).

### The link between organ weight and lipid metabolism in plants

Arabidopsis KLUH is proposed to produce an unknown signaling molecule that non-cell-autonomously regulates cell proliferation in different organs (Anastasiou *et al.*, 2007; Adamski *et al.*, 2009; Eriksson *et al.*, 2010). It has been hypothesized that the unknown signaling molecule might be fatty acid-derived molecules (Anastasiou *et al.*, 2007; Eriksson *et al.*, 2010; X. Sun *et al.*, 2017) based on the following evidence: (i) Arabidopsis CYP78A5, CYP78A7, and CYP78A10, and maize PLA1 hydroxylate short-chain fatty acids, including lauric acid (C12:0), myristic acid (C14:0), myristoleic acid (C14:1), and palmitic acid (C16:0) (Imaishi *et al.*, 2000; Kai *et al.*, 2009). Similarly, activation of rice CYP78A13 decreases nicotinic acid, shikimic acid, and quinic acid contents and increases the contents of glyceric acid and palmitic acid (Xu *et al.*, 2015). These results suggest that OsCYP78A13 might control organ growth via modification of short-chain fatty acid-derived molecules. Furthermore, OsCYP78A13 rescued the *klu-4* mutant, implying that the signals produced by the CYP78A subfamily proteins are identical in rice and Arabidopsis (Yang *et al.*, 2013; Xu *et al.*, 2015). However, the application of exogenous 12-hydroxylated lauric acid did not rescue the major phenotype of the *cyp78a5/a7* double mutant (Kai *et al.*, 2009), suggesting that the substrates catalyzed by CYP78A subfamily proteins remain elusive in plants. (ii) Cytochrome P450s catalyze various types of oxygenation reactions using fatty acids as substrates (Pinot and Beisson, 2011). Eight cytochrome P450 genes involved in fatty acid modification are transcriptionally regulated by KLUH/CYP78A5 activity in Arabidopsis (Anastasiou *et al.*, 2007). (iii) Many studies revealed a mechanistic link between lipid metabolism and seed size. For example, overexpression of *miRNA167A* results in lower α-linolenic acid content and larger seed size via decreased transcription of *fatty acid desaturase3* (*CsFAD3*) in *Camelina sativa* (Na *et al.*, 2019). Similarly, *GmFAD3*-silenced plants contain reduced levels of linolenic acid (18:3) and produce significantly larger seeds in soybean (Singh *et al.*, 2011). Down-regulation of *BnDof5.6* in canola reduces both embryo size and fatty acids content (Deng *et al.*, 2015). Moreover, reduced expression of *HECT E3 ligase* in canola results in larger seeds with increased lipid content (Miller *et al.*, 2019). Other studies have also supported a link between lipid metabolism and seed weight (Chen *et al.*, 2012; Liu *et al.*, 2016; Lunn *et al.*, 2018; Meru *et al.*, 2018; Guo *et al.*, 2019). For example, the mutation of Arabidopsis *TRANSPARENT TESTA2* (*TT2*) significantly increased the seed fatty acid content and decreased seed weight (Chen *et al.*, 2012). Overexpression of rice *ACYL-CoA-BINDING PROTEIN 2* (*OsACBP 2*) confers an increase in grain size and seed oil content (Guo *et al.*, 2019). Similarly, the increased seed oil is also concomitant with an increase in seed weight in transgenic lines overexpressing Arabidopsis *Seipin1* (*AtSEI1*) (Lunn *et al.*, 2018).

In the present study, the *fw3.2* NILs and the *RNAi-2Q1* that down-regulate the expression of *SlKLUH* offer opportunities to reveal the molecular mechanisms controlling fruit and seed weight by this CYP78A member. DEGs between the two expression datasets, the NILs and the *fw3.2*(*ys*)–*RNAi-2Q1*, were enriched for genes that were part of several lipid metabolism pathways (Figs 3D, 4C; Supplementary Fig. S6). These results provide an indication of a possible link between SlKLUH-mediated fruit and seed weight regulation and lipid metabolism in tomato such that decreased expression of *SlKLUH* results in increased expression of many lipid metabolism-related genes. In addition, the transgenic lines overexpressing *SlSHN1* that show a significant decrease in fruit and seed weight also indicate a correlation between fruit and seed weight and lipid metabolism.

In our study, we found that fruit and seed weight were significantly different between *cd2* and the control under field conditions and in opposite directions (Supplementary Fig. S12B). Furthermore, the lack of consistency between field and greenhouse and the differing response suggest that *CD2* has no dramatic effect on fruit and seed weight in tomato (Supplementary Fig. S12). Moreover, altered expression of other lipid metabolism-related genes such as *GDSL1* (*Solyc11g006250*) and *GLYCEROL-3-PHOSPHATE ACYLTRANSFERASE 6* (*SlGPAT6*, *Solyc09g014350*) have no demonstrable effect on fruit weight (Girard *et al.*, 2012; Petit *et al.*, 2016). These results indicate the complicated relationship between lipid metabolism and fruit and seed weight. Furthermore, co-expression analyses also did not show a tight link between *SlKLUH* and lipid metabolism. In fact, only 37 (~2.2%) and 44 (~3.5%) lipid metabolism-related genes were identified in YYM and WGM (Supplementary Table S5), respectively. Therefore, it is possible that lipid metabolism is associated with but not directly regulated by *SlKLUH*, and that this pathway instead is associated with photosynthesis-related processes. However, little is known about the relationships among KLUH, photosynthesis-related processes, and lipid metabolism, which need to be further studied.

### DEG and co-expression network analyses provide a valuable resource of candidate genes putatively involved in organ weight regulation in tomato and other plants

The molecular mechanisms underpinning KLUH-mediated fruit and seed weight are poorly understood in tomato. The differential expression and co-expression analyses led to the identification of a number of candidate genes putatively involved in organ weight regulation mediated by SlKLUH in tomato.

In addition to the nine TFs which are common DEGs in both the *fw3.2*(*ys*)–*fw3.2*(*wt*) and the *fw3.2*(*ys*)–*RNAi-2Q1* datasets (Supplementary Table S4), many DEGs encoding enzymes or transporters in ‘Cutin synthesis and transport’ and ‘Fatty acid elongation and wax biosynthesis’ pathways were also identified (Figs 5, 6). Moreover, some of them are putatively involved in both plant development and lipid metabolism based on previous studies, including *HOTHEAD* (*HTH*; *AT1G72970*) (*Solyc06g062600*), *DEFECTIVE IN CUTICULAR RIDGES* (*DCR*; *AT5G23940*) (*Solyc03g025320*), *ATP-BINDING CASSETTE G11* (*ABCG11*; *AT1G17840*) (*Solyc01g105450*), and lipid transfer protein (LTP) genes. HTH, catalyzing the biosynthesis of long-chain α-,ω-dicarboxylic fatty acids, is required for the prevention of organ fusions in floral organs in Arabidopsis and rice (Krolikowski *et al.*, 2003; Kurdyukov *et al.*, 2006; Akiba *et al*., 2013; Xu *et al.*, 2017). DCR is involved in cutin and triacylglycerol biosynthesis (Panikashvili *et al.*, 2009; Rani *et al.*, 2010). The *dcr* mutants had wider and longer seeds than the wild type (Rani *et al.*, 2010). ABCG11 is involved in sterol/lipid homeostasis and vascular development in addition to plant growth (Panikashvili *et al.*, 2010, 2011; Le Hir *et al.*, 2013; Yadav *et al.*, 2014). Seven out of eight *LTP* genes were down-regulated in *RNAi-2Q1* compared with *fw3.2*(*ys*) (Fig. 6). LTPs are known to affect cuticle biosynthesis and transport, as well as seed development (Kim *et al.*, 2012; Wang *et al.*, 2015*b*; Deng *et al.*, 2016; Kouidri *et al.*, 2018). Notably, tomato *CUTIN SYNTHASE1* (*SlCUS1*; *Solyc11g006250*), an important gene involved in the ‘Cutin synthesis and transport’ pathway, was up-regulated in 7 DPA pericarp of *fw3.2*(*wt*) and *RNAi-2Q1* (Fig. 5). It encodes GDSL-motif esterase/acyltransferase/lipase protein and has been shown to be associated with both lipid metabolism and epidermal cell development (Segado *et al.*, 2020).

In Arabidopsis, nine cytochrome P450 genes were transcriptionally affected by mutations in CYP78A5/KLUH (Anastasiou *et al.*, 2007) of which eight were linked to fatty acid modifications. In our datasets, only *CYP76C4* (*Solyc02g090350*, *AT2G45550*) was identified as a down-regulated DEG in 7 DPA pericarp and seeds in *RNAi-2Q1* (Supplementary Dataset S3), but not in the NILs, suggesting a role for *CYP76C4* in the KLUH pathway in both tomato and Arabidopsis. Further studies are required to confirm the biological and biochemical functions of *CYP76C4* and its relationship to KLUH in Arabidopsis and tomato.

Co-expression network analyses identified common and unique gene sets between YYM and WGM including many TFs putatively associated with fruit and seed weight in tomato (Supplementary Table S4). Co-expressed genes of interest that are not TF genes in YYM and WGM were also found. For example, the RING-type E3 ubiquitin ligase *EOD1* (*AT3G63530*) (*Solyc11g062260*) was identified as a negative regulator of organ size (Disch *et al.*, 2006; Li *et al.*, 2008; Li and Li, 2015; Vanhaeren *et al.*, 2017). The *eod1* mutants had larger organs and increased biomass, while overexpression of *EOD1* resulted in reduced organ growth (Disch *et al.*, 2006; Xia *et al.*, 2013). Arabidopsis *DWF4* (*AT3G50660*) (*Solyc02g093540*) encodes a C-22 hydroxylase that catalyzes a rate-determining step in brassinosteroid biosynthesis. Overexpression of *DWF4* significantly increased seed number and weight, thus increasing seed yield in Arabidopsis (Choe *et al.*, 2001), *Brassica napus* (Sahni *et al.*, 2016), and rice (Wu *et al.*, 2008).

Together, we propose many DEGs and co-expressed genes that are putatively involved in the fruit and seed weight regulation mediated by SlKLUH. This knowledge is helpful to elucidate the whole picture of the KLUH pathway regulating organ size in tomato and other crops. However, the exact functions of these candidate genes remain to be studied in tomato.

### Conclusion

**Fig. 12.**
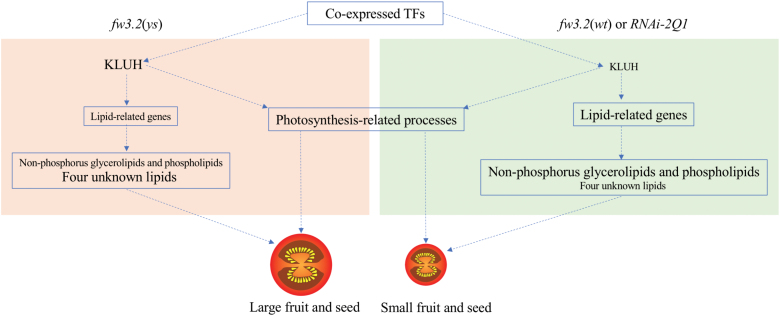
Proposed model of *SlKLUH*-mediated regulation of fruit and seed weight in tomato. The differential expression of *SlKLUH* results in altered expression of many lipid-related genes, photosynthesis-related processes, and lipid profiles that can determine genotype-specific fruit and seed size. The font size of ‘KLUH’ and ‘Lipid-related genes’ corresponds to the expression levels. The font size of ‘Non-phosphorus glycerolipids and phospholipids’ and ‘Four unknown lipids’ indicates the contents of the lipids.

Our results reinforce the notion that lipid metabolism is involved in SlKLUH-mediated regulation of fruit and seed weight through a possible mechanism as proposed in Fig. 12. The differential expression of *SlKLUH* between the NILs results in different co-expression networks that are associated with fruit and seed development, possibly through modulating photosynthesis-related processes. The TFs identified in YYM and WGM are putative upstream regulators of *SlKLUH*. In the small-fruited NIL *fw3.2*(*wt*) and *RNAi-2Q1*, lower expression of *SlKLUH* is associated with increased expression of many genes involved in lipid metabolism, especially for genes involved in ‘Cutin synthesis and transport’ and ‘Fatty acid elongation and wax biosynthesis’. Thus, the contents of certain non-phosphorus glycerolipids and phospholipids were increased while the contents of the four unknown lipids were decreased. Importantly, a number of lipid-related genes and TFs putatively involved in the regulation of fruit and seed weight in tomato were also identified, providing potential targets for further dissecting the molecular mechanisms underlying fruit and seed weight in tomato and other crops.

## Supplementary data

The following supplementary data are available at *JXB* online.

Table S1. Summary of RNA-seq mapping for all samples.

Table S2. Shared DEGs in 7 DPA pericarp and seed in *fw3.2*(*ys*)–*fw3.2* (*wt*) and *fw3.2*(*ys*)–*RNAi-2Q1* comparisons.

Table S3. Lipid-related DEGs in the *fw3.2*(*ys*)–*fw3.2*(*wt*) and *fw3.2*(*ys*)–*RNAi-2Q1* comparisons.

Table S4. Common transcription factors identified in *fw3.2*(*ys*)–*fw3.2*(*wt*) and *fw3.2*(*ys*)–*RNAi-2Q1* comparisons and transcription factors in YYM and WGM.

Table S5. Lipid-related genes in YYM and WGM.

Fig. S1. Developing fruit at six developmental time points in the *fw3.2* NILs.

Fig. S2. Phenotypic evaluations of the NILs in the second replication.

Fig. S3. Spearman correlation coefficient (SCC) analysis of transcriptomic profiles of the 48 replicates from *fw3.2* (*ys*) and *fw3.2*(*wt*).

Fig. S4. Spearman correlation coefficient (SCC) of transcriptomic profiles of all 12 replicates from transgenic lines *RNAi-2G2* and *RNAi-2Q1* that down-regulate *SlKLUH*.

Fig. S5. Up- and down-regulated genes in pericarp and seed at 7 DPA in the *RNAi-2Q1* or *fw3.2*(*wt*) compared with the *fw3.2* (*ys*).

Fig. S6. Overview of the distribution of the DEGs in lipid metabolism pathways.

Fig. S7 Comparison of known lipids of 5 DPA fruits from *fw3.2* (*ys*), *fw3.2*(*wt*), and *RNAi-2Q1*.

Fig. S8. Comparison of co-expressed genes in YYM and WGM.

Fig. S9. Expression profiles of *Solyc04g074990* (ZF-HD), *Solyc08g079800* (GRF), and *Solyc04g077510* (GRF) in developing pericarp and seeds in the NILs.

Fig. S10. Overview of distribution of TF families that were co-expressed with *SlKLUH* in YYM and WGM.

Fig. S11. Phylogenetic analysis of AP2 transcription factors from tomato, Arabidopsis, and five WRI1 orthologs from other plant species.

Fig. S12. Fruit weight and 50 seeds weight of *cd2* and Ailsa Craig (AC) control from the greenhouse and field trial.

Dataset S1. DEGs at different developmental time points of pericarp and seed in the *fw3.2* NILs.

Dataset S2. DEGs significantly affected by genotype in the *fw3.2* NILs.

Dataset S3. DEGs in 7 DPA pericarp and seed between *fw3.2*(*ys*) and *RNAi-2Q1*.

Dataset S4. Co-expressed genes in the 11 modules identified in *fw3.2*(*ys*) using WGCNA.

Dataset S5. Co-expressed genes in the 10 modules identified in *fw3.2*(*wt*) using WGCNA.

eraa518_suppl_Supplementary-Datasets-S1-S5

eraa518_suppl_Supplementary-Figures-S1-S12

eraa518_suppl_Supplementary-Tables-S1-S5

## Data Availability

The data supporting the findings of this study are available from the corresponding author, EvdK, upon request.
