# Supplementary material for: Differential expression of SlKLUH controlling fruit and seed weight is associated with changes in lipid metabolism and photosynthesis-related genes
Source: J Exp Bot. 2020 Nov 7;72(4):1225–44. doi: 10.1093/jxb/eraa518 (PMC7904157; doi:10.1093/jxb/eraa518)
Supplement: eraa518_suppl_Supplementary-Figures-S1-S12 [file eraa518_suppl_supplementary-figures-s1-s12.pdf]

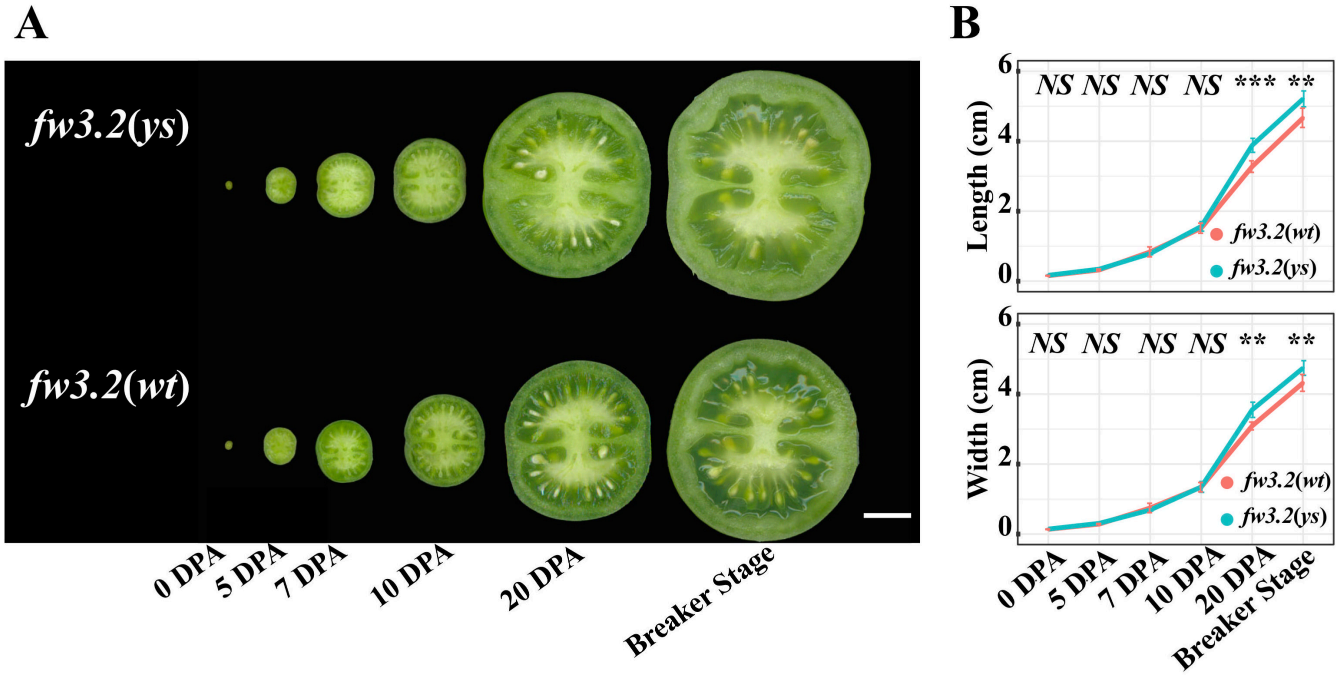

Fig. S1 Developing fruit at six developmental timepoints in the *fw3.2* NILs. (A) Medio-lateral section of fruits at different stages of development. Scale bar = 1 cm; (B) Fruit size comparison showing variation in fruit length and width of *fw3.2*(*ys*) and *fw3.2*(*wt*) at different developmental timepoints. Asterisks denote significant differences (**, $P < 0.01$; ***, $P < 0.001$) as determined by Student's t-tests. DPA, days post anthesis. *NS*, non-significant difference.

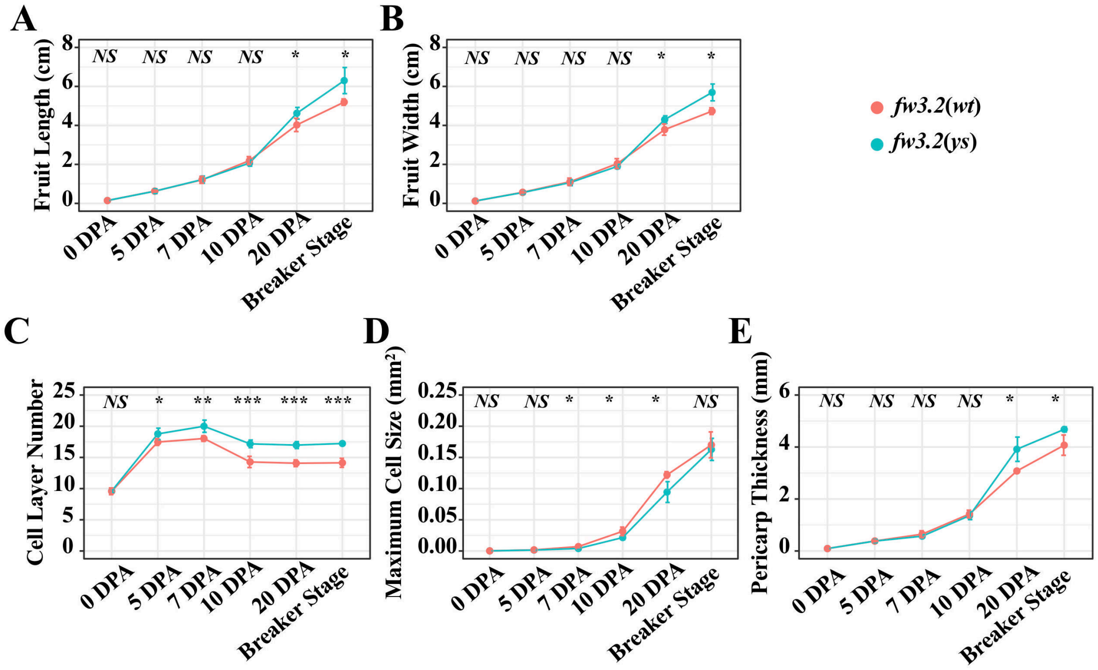

Fig. S2 Phenotypic evaluations of the NILs in the second replication. Asterisks denote significant differences (*, P < 0.05; **, P < 0.01; ***, P < 0.001) as determined by Student's t-tests. DPA, days post anthesis. NS, non-significant difference.

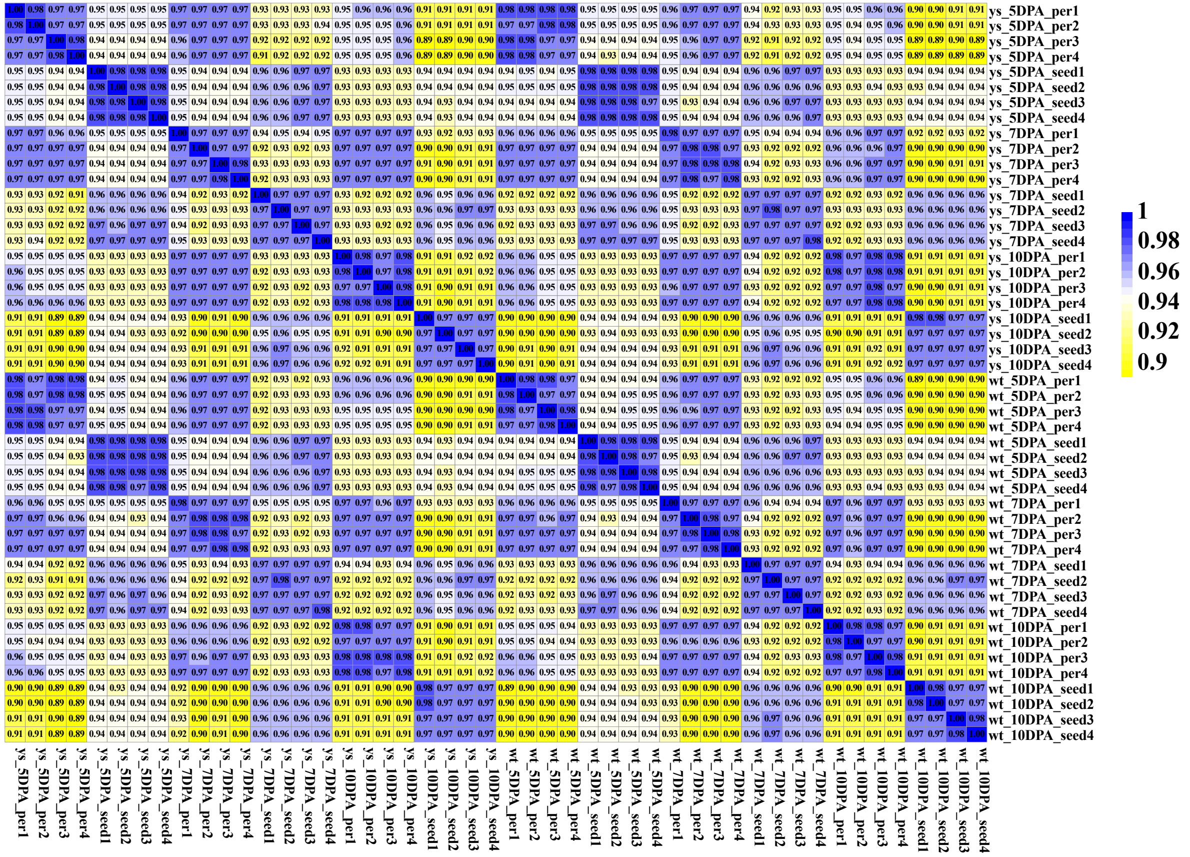

Fig. S3 Spearman correlation coefficient (SCC) analysis of transcriptomic profiles of the 48 replicates from *fw3.2* (*ys*) and *fw3.2*(*wt*). Heatmap shows high correlation (0.97-0.98) among biological replicates. ys, *fw3.2* (*ys*); wt, *fw3.2*(*wt*); per, pericarp.

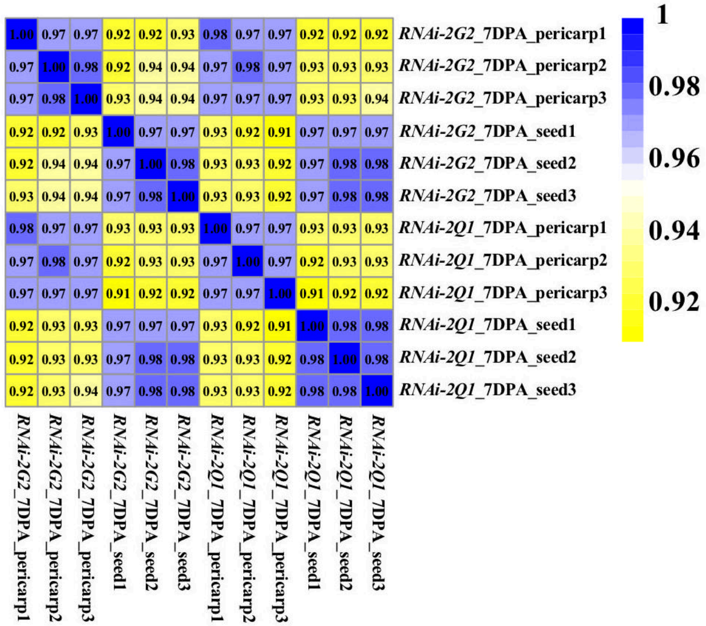

Fig. S4 Spearman correlation coefficient (SCC) of transcriptomic profiles of all 12 replicates from transgenic lines *RNAi-2G2* and *RNAi-2Q1* that down regulate *SlKLUH*. Heatmap shows high correlations (0.97-0.98) among biological replicates.

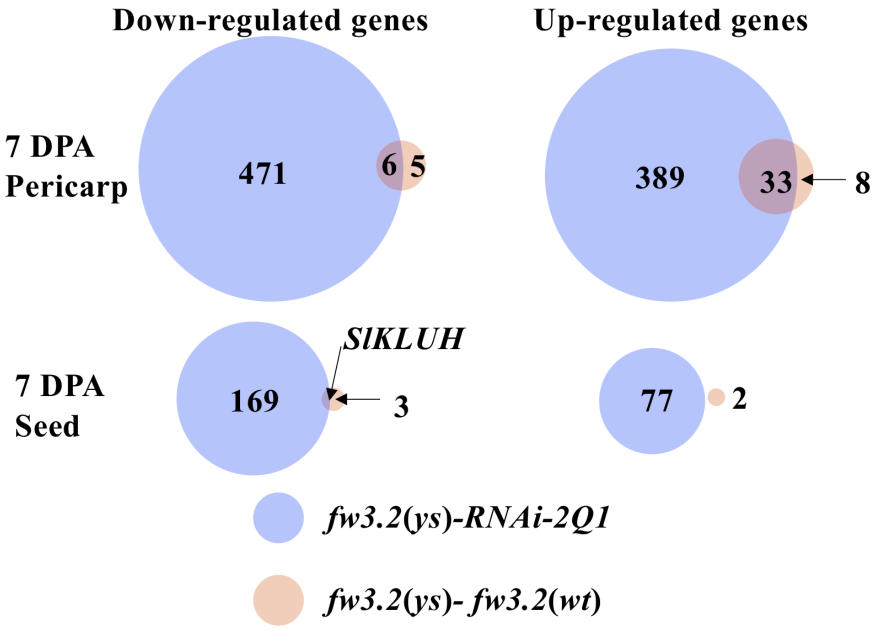

Fig. S5 Up- and down-regulated genes in pericarp and seed at 7 DPA in the *RNAi-2Q1* or *fw3.2*(*wt*) compared to the *fw3.2* (*ys*). The circle areas are proportional to gene number.

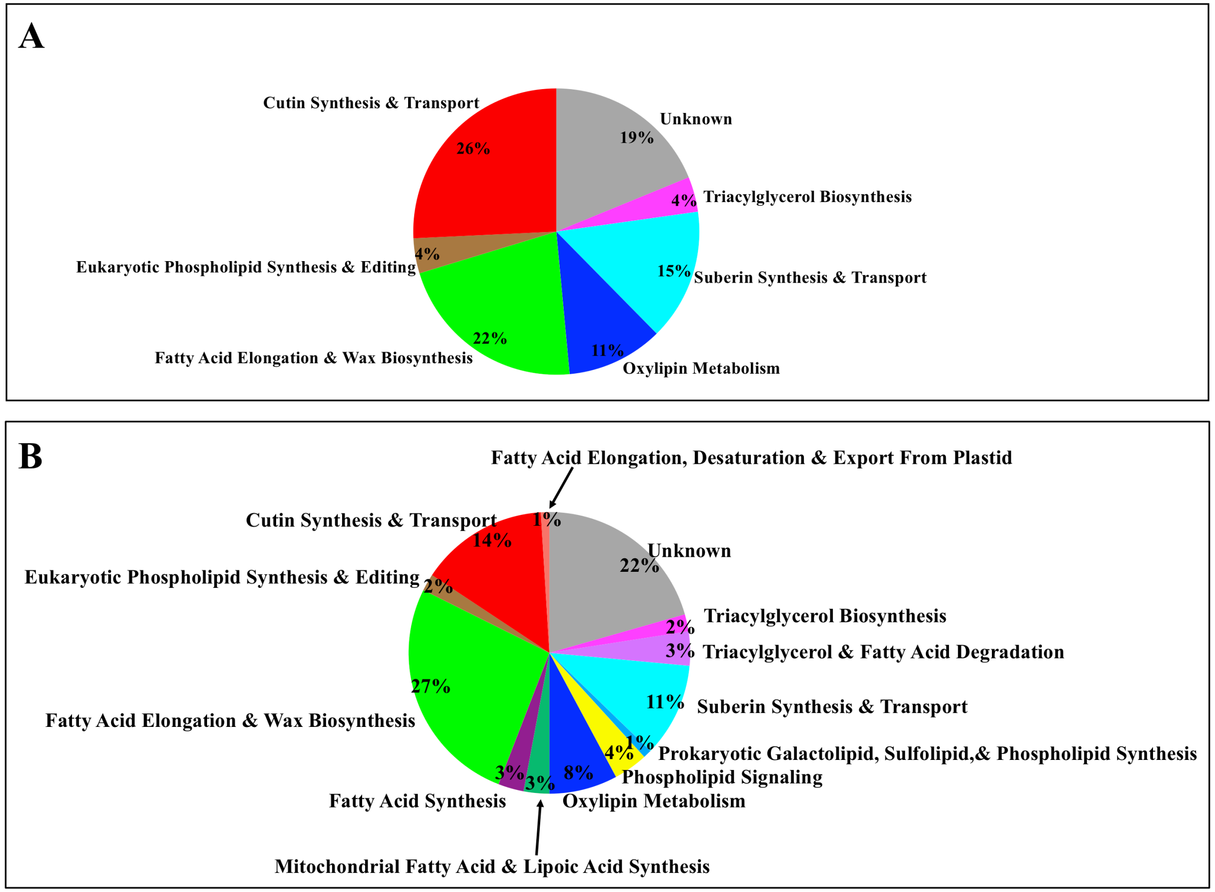

Fig. S6 Overview of distribution of the DEGs in lipid metabolism pathways. (A) Lipid metabolism-related DEGs between *fw3.2*(*ys*) and *fw3.2*(*wt*); (B) Lipid metabolism-related DEGs between *fw3.2*(*ys*) and *RNAi-2Q1*.

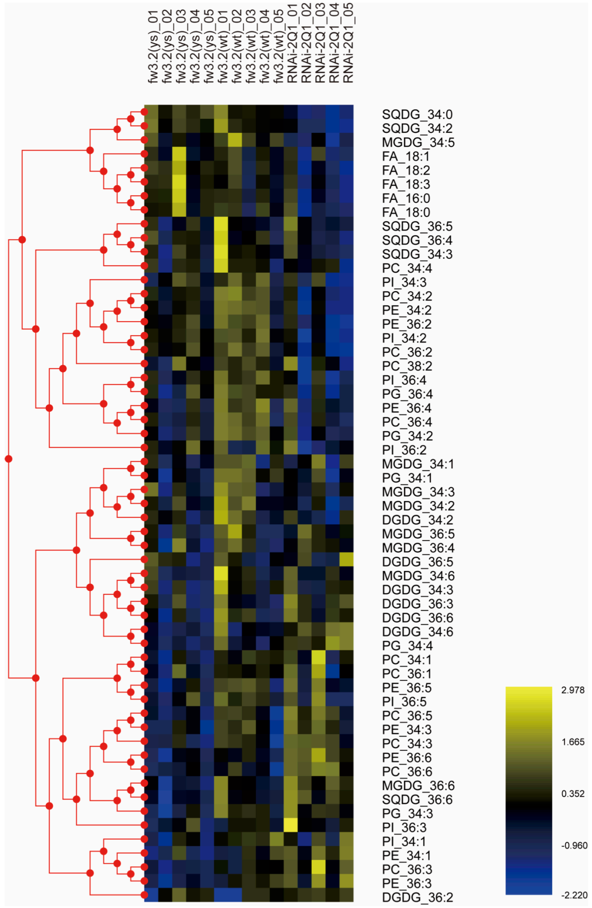

Fig. S7 Comparison of known lipids in 5DPA fruits from *fw3.2* (*ys*), *fw3.2*(*wt*) and *RNAi-2Q1*. Each colored bar within a column represents an ion detected by LC-Q-tof MS in the negative ion mode. Peak picking and alignments were performed using Waters Masslynx. The peak height of each metabolite was transformed to a z-score. The heatmap accumulation and hierarchical clustering analysis to find the series of metabolites whose abundance changed simultaneously was done using the WebMeV platform (http://mev.tm4.org/).

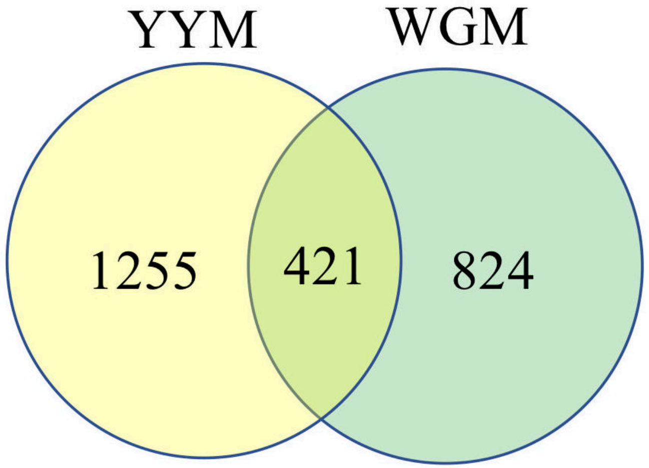

Fig. S8 Comparison of co-expression genes in YYM and WGM.

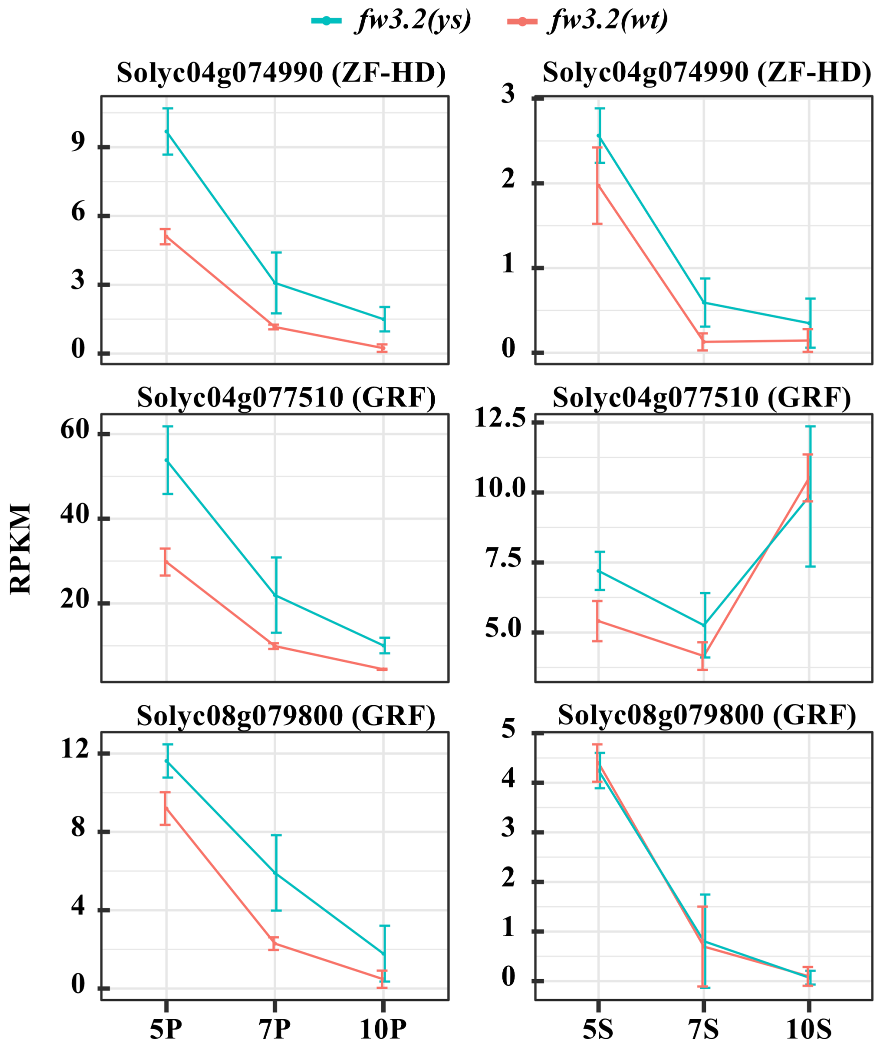

Fig. S9 Expression profiles of *Solyc04g074990* (ZF-HD), *Solyc08g079800* (GRF) and *Solyc04g077510* (GRF) in developing pericarp and seeds in the NILs. The numbers 5, 7 and 10 indicate 5 DPA, 7 DPA and 10 DPA, respectively. P, pericarp; S, seed.

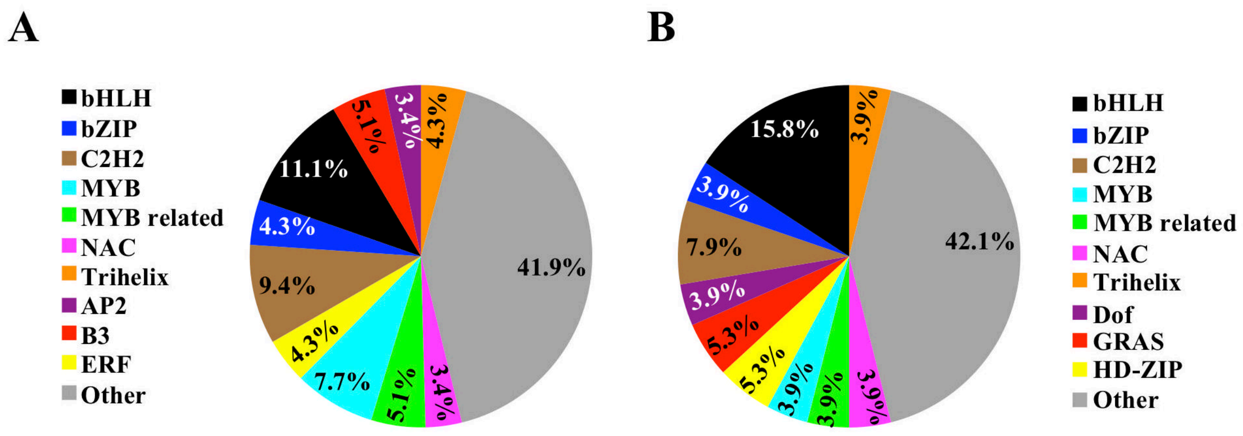

Fig. S10 Overview of distribution of TF families that co-expressed with *SlKLUH* in YYM (A) and WGM (B).

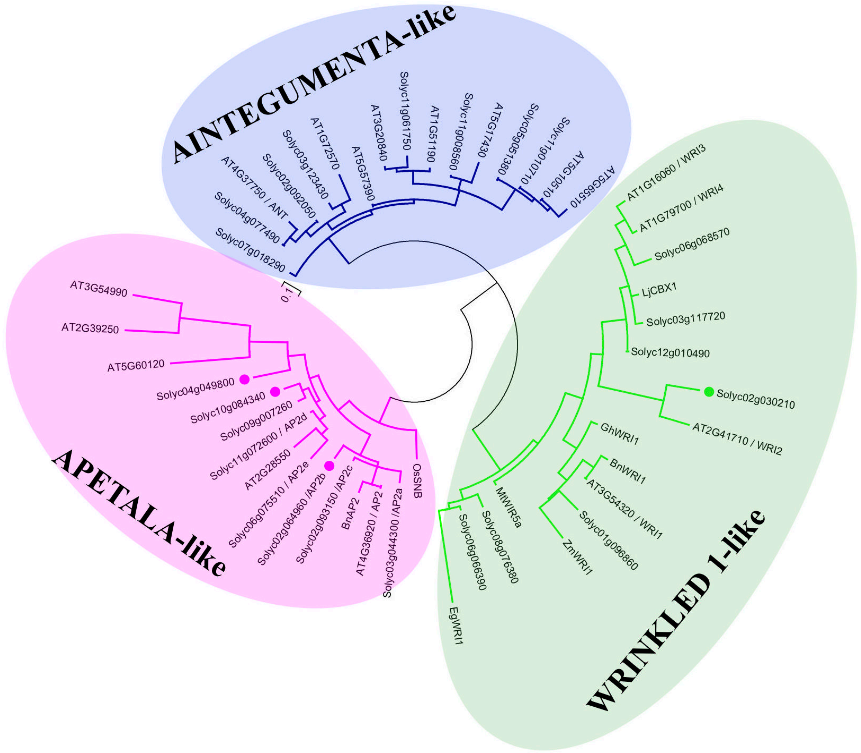

Fig. S11 Phylogenetic analysis of AP2 transcription factors from tomato, Arabidopsis and five WRI1 orthologs from other plant species. The alignment of protein sequences was performed using ClustalX 1.81, and the phylogenetic tree was constructed by MEGA7 using the Maximum Likelihood algorithm. Green and pink dots indicate co-expressed genes of *SlKLUH* in YYM. Green dot indicates closest ortholog of AtWRI1 in tomato; pink dots indicate three closest orthologs of AtAP2 in tomato. The three clades WRINKLED1-like, APETALA-like, and AINTEGUMENTA-like are highlighted by different background colors.

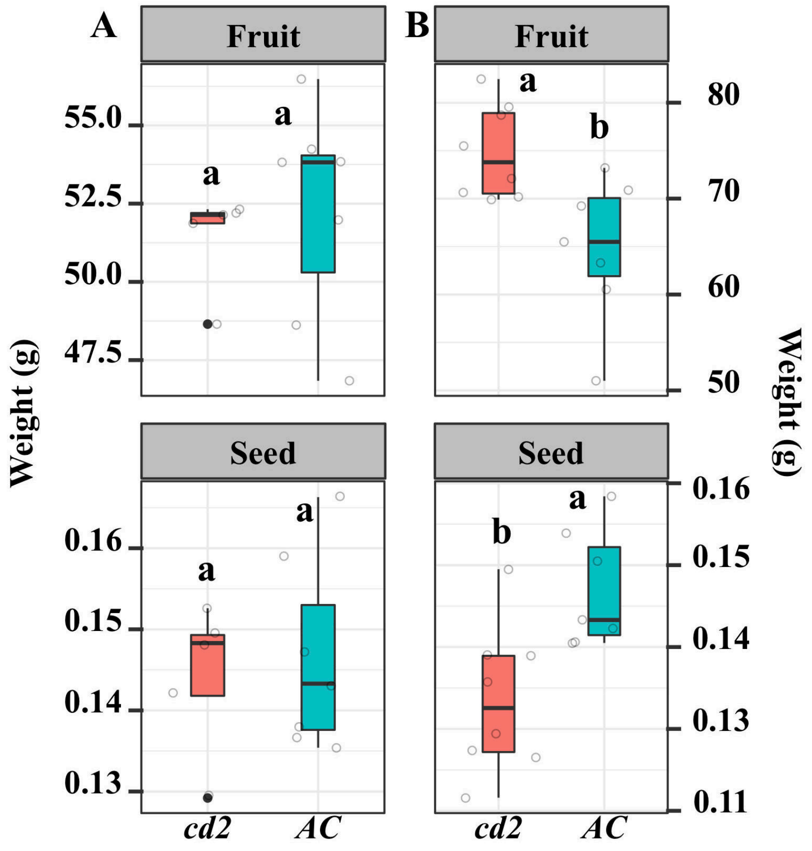

Fig. S12 Fruit weight and 50 seeds weight of *cd2* and Ailsa Craig (AC) control from greenhouse (A) and field trial (B). The letters in the boxplots indicate the significant differences among different genotypes evaluated by Duncan's test ($\alpha < 0.05$).
